# Solution-processable microporous polymer platform for heterogenization of diverse photoredox catalysts

**DOI:** 10.1038/s41467-022-29811-6

**Published:** 2022-05-27

**Authors:** Richard Y. Liu, Sheng Guo, Shao-Xiong Lennon Luo, Timothy M. Swager

**Affiliations:** 1 Institute for Soldier Nanotechnologies, 500 Technology Square, Cambridge, MA 02139 USA; 2 Department of Chemistry, 77 Massachusetts Avenue, Cambridge, MA 02139 USA

**Keywords:** Flow chemistry, Photocatalysis

## Abstract

In contemporary organic synthesis, substances that access strongly oxidizing and/or reducing states upon irradiation have been exploited to facilitate powerful and unprecedented transformations. However, the implementation of light-driven reactions in large-scale processes remains uncommon, limited by the lack of general technologies for the immobilization, separation, and reuse of these diverse catalysts. Here, we report a new class of photoactive organic polymers that combine the flexibility of small-molecule dyes with the operational advantages and recyclability of solid-phase catalysts. The solubility of these polymers in select non-polar organic solvents supports their facile processing into a wide range of heterogeneous modalities. The active sites, embedded within porous microstructures, display elevated reactivity, further enhanced by the mobility of excited states and charged species within the polymers. The independent tunability of the physical and photochemical properties of these materials affords a convenient, generalizable platform for the metamorphosis of modern photoredox catalysts into active heterogeneous equivalents.

## Abstract (web summary)

Heterogeneous photocatalysts are easily separable and recyclable, but homogeneous ones are precisely tunable. Here, the authors merge the benefits of these two catalyst types by incorporating diverse dyes into solution-processable, porous polymers.

## Introduction

Photoredox catalysis refers to an energy-conversion process in which the absorption of photons by a substance triggers electron transfer. In the past decade, the application of this phenomenon to organic synthesis has evolved from an academic curiosity to an enabling technology widely adopted in pharmaceutical synthesis^1–7^. By coupling photoexcitation with other molecular processes, including cooperative catalytic schemes^8–10^, hundreds of powerful and previously inconceivable organic transformations have now been realized. However, compared to the rapid discovery of new photon-dependent reactions, the implementation of these processes on large scale has seen only limited progress due to the high cost of many photocatalysts, challenges in catalyst removal, and restricted light penetration in traditional batch reactors^11^. Thus, the conception of new technologies^12–15^ that generally enhance photoredox catalysis on scale is a pressing goal that has attracted immense research effort.

**Fig. 1:**
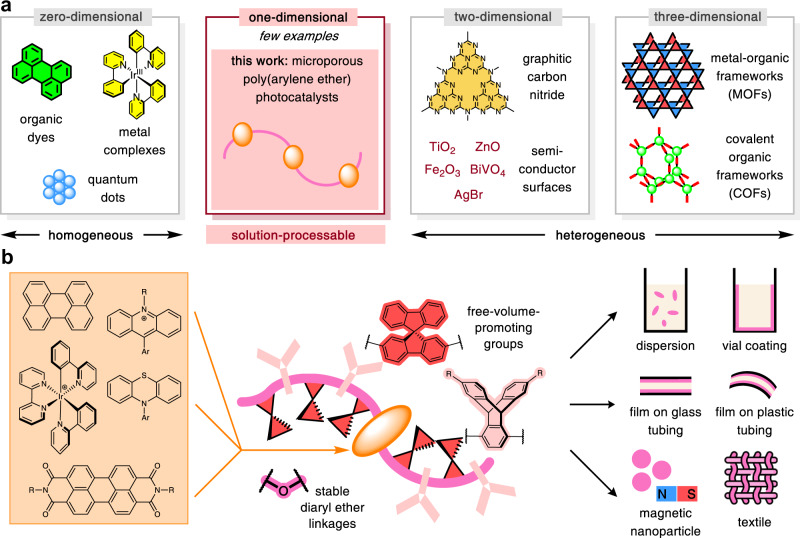
Overview of photoredox catalyst design. **a** Classification of photoredox catalysts according to solubility and geometric dimension of active surface. **b** Transforming a broad scope of small-molecule photocatalysts into diverse heterogeneous materials through solution-processable polymers.

Photoredox catalysts can be divided into two major groups (Fig. 1a): homogenous materials, which are soluble in the solvents typically used for photoredox catalysis, and heterogeneous, which operate as insoluble solids. Due to ease of synthesis and tunability, the vast majority of newly developed catalysts belong to the former group, which includes small-molecule organic dyes^3^, such as perylene diimides (PDIs), and organometallic complexes^5^, such as Ir(ppy)_3_. Meanwhile, heterogeneous catalysts have the advantages of facile removal from reaction mixtures and recyclability, which are important from both cost and sustainability perspectives^6,11^. Well-known examples include two-dimensional (2-D) surface photocatalysts, especially semiconductors^16^ such as mesoporous graphitic carbon nitride (mpg-CN)^17^, and porous three-dimensional (3-D) constructs, such as metal-organic/covalent organic frameworks (MOFs/COFs)^18–20^. The appealing characteristics of these materials arrive at the expense of diversity and tunability: there is often no rational technique to finely alter their photochemical properties while retaining the bulk physical properties. Importantly, the inability of most heterogeneous catalysts to be cast into films or coatings from liquid or solution phase is a considerable practical limitation, particularly towards their synergistic application with emerging technologies such as continuous-flow synthesis^10,11^.

A strategy that allows synthetic chemists to simultaneously take advantage of the respective desirable properties of homogeneous and heterogeneous catalysts would be exceptionally empowering. We proposed that one solution might involve a general method for converting any member of the vast toolbox of soluble photocatalysts into equivalent insoluble materials, while maintaining the original photochemical attributes and activity. However, to our knowledge, no heterogenization approach exists with broad scope both in the kinds of dyes incorporated and in the types of catalytic materials accessible. Although several examples of photocatalyst immobilization on insoluble solids have been demonstrated^21^, each strategy addresses only a small subset of chromophore structures, and the vast majority of supported catalysts lack solution-processability, severely limiting the operational modes that are available (typically only particles or dispersions).

Herein, by extending our recently reported Pd-catalyzed polycondensation reaction^22^, we present a method that, in principle, allows for any organic or organometallic photocatalyst to be heterogenized through copolymerization with porosity-promoting bulky organic monomers (triptycenes and spirobifluorenes)^23–25^. We note that while 2D and 3D extended materials are well represented among heterogeneous photocatalysts, examples of one-dimensional (1D) photocatalysts, in which the active components form chains, are relatively rare^26,27^. Importantly, our linear-polymer structures, while insoluble in the solvents typically employed for photoredox catalysis, remain highly soluble in a few, select handling solvents (DCM and THF). As a result of this processability, our immobilization technique is not restricted to any particular target mode of heterogeneous catalysis but can conveniently access a wide range of known morphologies, including dispersions, films, coatings, textiles, and magnetic nanoparticles.

Aside from solution-processability and broad generality, our 1D polyether platform offers additional valuable benefits compared to existing heterogeneous photoredox catalysts. First, the solubility of organic polymers enables simple characterization of structure, molecular weight distribution, photocatalyst loading, defect concentration, and batch-to-batch quality variations, all of which are often impractical to analyze for conventional insoluble catalysts. Second, traditional materials relying on non-covalent interactions for dye attachment, or labile covalent bonds such as Si–O or Al–O bonds, can exhibit poor chemical stability^21^. In contrast, the polymers developed in this work exhibit exceptional thermal stability and inertness toward a variety of chemical conditions. Third, the immobilization of small-molecule catalysts on heterogeneous supports often leads to drastically decreased turnover frequencies; however, with our polymer materials, due to a combination of reduced aggregation-induced quenching, highly porous-polymer structure, and energy- and charge-transport processes, very efficient catalysis can be achieved, in some cases with rates exceeding than with the corresponding monomers. Finally, the design of our polymers enables independent control of the physical and photochemical properties without alteration to the synthetic procedure.

## Results and discussion

**Fig. 2:**
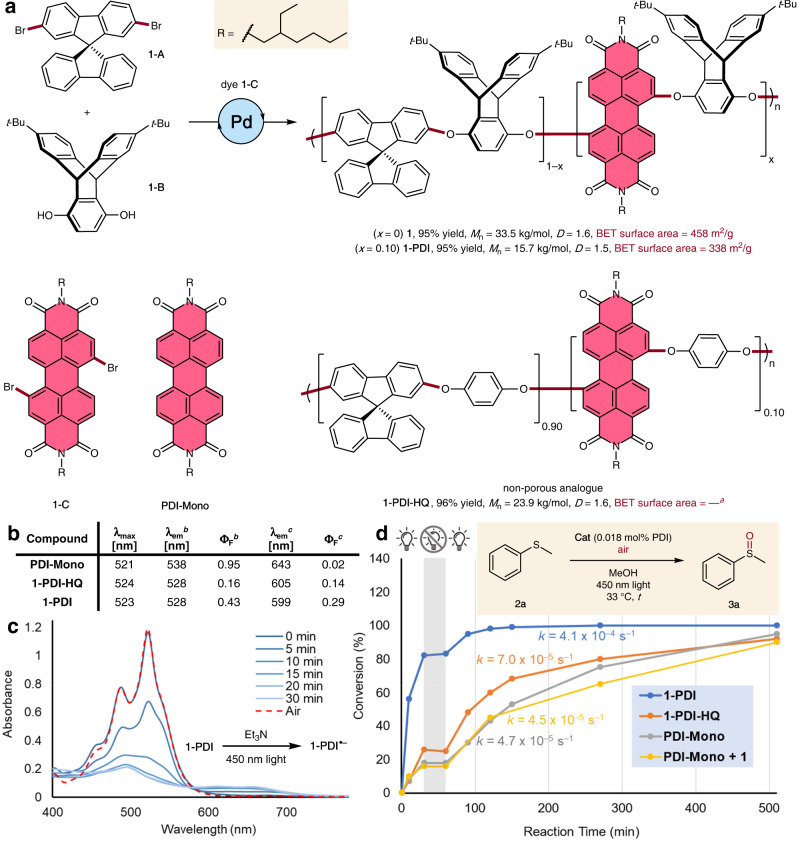
Perylene diimide porous-polymer catalyst. **a** Synthesis of **1**, **1-PDI**, and **1-PDI-HQ**. See the Supplementary Information for detailed conditions. **b** Photophysical properties. **c** Light-driven reversible redox process with an amine partner. **d** Kinetics of catalytic photo-oxidation model reaction. BET = Brunauer–Emmett–Teller method. ^a^Too low to be reliably determined from N_2_ adsorption isotherm. ^b^Dilute THF solution (1.0 × 10^–5^–5.0 × 10^–7^ M). ^c^Solid state (powder).

Our recent systematic study^22^ showed that the foundational building blocks of our proposed material, *t*-Bu-triptycene hydroquinone **1-A** and spirobifluorene dibromide **1-B**, could be cross-coupled to form polymer **1** with excellent molecular weight, polydispersity, porosity, and film properties (Fig. 2a). Through slight alteration of these prior conditions, we were able to introduce chromophore-containing comonomers to generate materials with similar physical attributes as **1**, but with added photocatalytic capabilities. As a model, we selected the classic dye perylene diimide (PDI) as synthetically versatile redox-active moiety^28^. With the addition of 10% of PDI dibromide (**1-C**) in the polycondensation process, we obtained a near-quantitative yield of a deep-red solid (**1-PDI**) with high molecular weight and moderate polydispersity. The porosity was evaluated through the Brunauer–Emmett–Teller (BET) gas adsorption method, revealing a considerable specific surface area (SSA) of 338 m^2^/g. The essential role of the triptycene units in endowing the polymer with this exceptional porosity is evident from the very low SSA of related polymer **1-PDI-HQ**, in which *para*-phenylenes are present instead. Based on the N_2_ adsorption isotherm, the estimated pore size distribution was concentrated in the microporous range (<2 nm, Supplementary Fig. 3-1). Furthermore, the polymer showed excellent thermal and chemical stability under controlled conditions (Supplementary Fig. 4-2). The extent of incorporation of the PDI was determined by ^1^H NMR analysis to be 10%, in agreement with the fraction of monomer employed during the synthesis.

The absorption and steady-state photoluminescence spectra of **1-PDI** contained distinctive features (λ_max_ = 523 nm, λ_em_ = 528 nm in THF solution) characteristic of PDI units (Fig. 2b). The solid-state photophysical properties of **1-PDI** showed notable differences when compared against monomeric (**PDI-Mono**) and non-porous (**1-PDI-HQ**) analogues. Although the molecular dye displayed a very low fluorescence quantum yield in the solid state (QY = 0.02), the chromophores covalently hosted within triptycene-containing frameworks were bright emitters (QY = 0.29 for **1-PDI**), indicating a reduction in aggregation-based quenching. Monomeric PDIs are known to exhibit a substantial emission red-shift upon aggregation^29^, and we found that the difference in emission maxima between solution and solid states was substantially smaller for the polymers than for monomers and, in particular, smallest for the porous **1-PDI**.

As a test of the photo-activated reactivity of these polymers, irradiation of a DCM solution of **1-PDI** with excess Et_3_N resulted in isosbestic conversion to a new species, with spectral features indicative of PDI radical anions (Fig. 2c)^30^. Exposure of the resulting solution to atmospheric oxygen regenerated the original polymer over the course of 2 h. Given its promising reversibly stoichiometric reactivity, a dispersion of the catalyst in methanol was evaluated in a simple aerobic photo-oxidation reaction^31^ of methyl phenyl sulfide (**2a**, Fig. 2d). At remarkably low loading of catalyst (0.018 mol%), full consumption of starting material could be observed after 6 h. Parallel experiments at equivalent optical densities of a non-porous analogue (**1-PDI-HQ**) and a monomeric PDI (**PDI-Mono**) proceeded at a significantly lower rate and only preceded to 80 and 76% conversion over the same time period. An additional control using a combination of **PDI-Mono** with dye-free polyether **1** (premixed in dichloromethane solution, then dried and dispersed in methanol) showed a reaction rate similar to using **PDI-Mono** alone. The reactions did not progress during a 30 min interval with no LED irradiation, confirming that the chosen transformation requires continuous photoirradiation. We hypothesized that, in addition to the steric insulation from aggregation-based quenching, the elevated activity of **1-PDI** is partially the result of the photoexcited state “hopping” between different dye centers, thereby enhancing the probability of encounter with a substrate or cocatalyst. Indeed, using fluorescence polarization spectroscopy, we observed quencher-dependent behavior consistent with considerable excited-state energy transfer between dye units (for extended discussion, see the Supplementary Information, Section 10.2, Figs. S7-2–S7-4). The mobility of charged states throughout the porous polymer can similarly contribute to an enhanced efficiency. In bulk conductivity measurements, we showed that irradiation of a film of **1-PDI** in the presence of triethylamine results in an instantaneous and reversible increase in conductivity (Supplementary Fig. 7-5), suggesting facile transport of negative charges between PDI units.

**Fig. 3:**
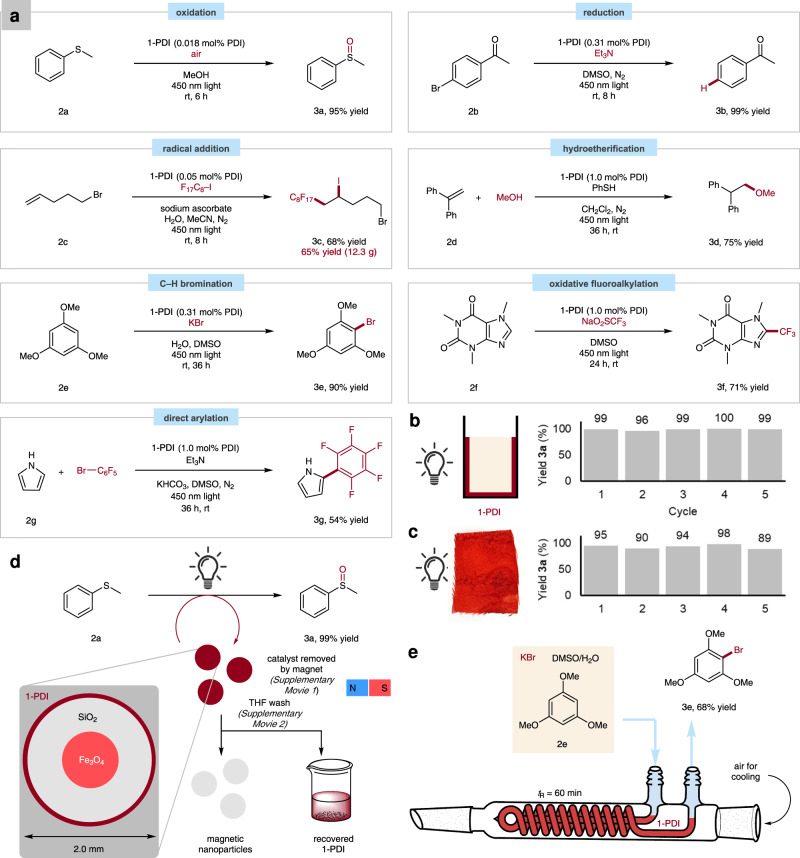
Synthetic applications of porous-polymer photocatalyst. **a** Performance of **1-PDI** across various reaction classes. **b** Recyclable usage of **1-PDI** as a coating on a glass vial in the photo-oxidation of **2a**. **c** Recyclable usage of **1-PDI** deposited on cotton in the photo-oxidation of **2a**. **d** Coated superparamagnetic silica nanoparticles. **e** Continuous-flow synthesis using a film of **1-PDI** in glass tubing.

To illustrate the synthetic versatility of porous linear-polymer catalysts, we applied **1-PDI** to a representative range of modern photoredox transformations (Fig. 3a). On a 1.0 mmol scale, the aerobic oxidation of thioether **2a** to sulfoxide **3a** was accomplished in 95% yield using an alcoholic dispersion of **1-PDI** and irradiation with a 450 nm LED. Photoreduction reactions could be performed through the in situ generation of PDI radical anion under inert atmosphere^29^. Using triethylamine as the terminal reductant, aryl bromide **2b** was successfully hydrodehalogenated. The same catalyst could induce atom-transfer radical addition reactions to alkenes: fluorinated alkyl bromide **3c** was prepared in high yield from 5-bromo-1-pentene (**2c**) and perfluorooctyl iodide^32^. Since **3c** is a non-commercial fluorosurfactant essential to other research in our laboratory, we pursued a scaled-up synthesis enabled by catalyst **1-PDI**. Over 12 g of product could be obtained in a single run, demonstrating the applicability of these porous materials in a preparative setting. Heterogeneous photoredox conditions were also applicable in polar addition to alkenes, as shown by the anti-Markovnikov hydroetherification of **2d**^33^. In another activation mode, the oxidative C–H functionalization of electron-rich arene **2e** was conducted using potassium bromide as the bromine-atom source^17^. Both oxidative and reductive modes of radical generation were successful using **1-PDI** as a photocatalyst, and regioselective addition of these radicals to heteroarenes was accomplished (**3f** and **3g**)^34,35^.

Many valuable modes of heterogeneous catalysis are accessible due to the solubility profile of poly(arylene ether)s. Using a standard rotary evaporator, a thin film layer could be deposited on the bottom portion of a glass reaction vial, which has maximal exposure to the light source (Fig. 3b). The coated vessel could be conveniently reused to perform the photo-oxidation of **2a**, with negligible change in reaction yield over five cycles. Textile-supported organocatalysts have recently been advanced as convenient promoters for acid- and base-dependent reactions^36^. We showed that **1-PDI** can be robustly deposited onto cotton, and that the resulting fluorescent fabric is also a reusable photo-oxidation catalyst (Fig. 3c). The polymer coating could adhere strongly to silica nanoparticles bearing a superparamagnetic iron oxide core (Fig. 3d), which facilitates separation from reaction mixtures for which filtration is impractical. The resulting red powder was an effective agent in photoredox reactions, and the application of a handheld permanent magnet allowed for rapid and complete separation of the catalyst from the methanol solution (Supplementary Movie 1). The catalytic layer could be instantly desorbed from the surface by immersion in tetrahydrofuran and thus recovered from the silica (Supplementary Movie 2).

Continuous-flow synthesis, referring to processes performed within narrow tubing under constant material input and output, has attracted significant attention as an alternative to traditional batch chemical processes^37,38^. Flow systems are associated with superior heat exchange, mixing, reproducibility, and safety profiles^39^. The narrow-channel configuration of the reactors has proved particularly promising in large-scale photocatalysis applications, as enhanced light exposure is afforded with decreased risk of over-irradiation that can induce unwanted side-reactions^40^. A solution-processable material for the immobilization of diverse photocatalysts along the interior surface of the reactor tubing would provide two main advantages. First, no separation of the dyes from the mobile phase would be required after the reaction. Second, by concentrating the chromophores close to the exterior of the reaction mixture, light exposure could be enhanced. Taking advantage of the excellent film-forming characteristics of our porous polyethers, we showed that flow chemistry could be conducted using a photoredox-active coating as a stationary phase (Fig. 3e). To the coolant coil of a spiral reflux condenser, a standard piece of equipment in laboratories that perform microscale organic synthesis, was applied a thin layer of **1-PDI** as a solution in THF, drying with gentle heating and air flow. With the system under constant irradiation with blue LEDs, reactants for the C–H bromination of **2e** were pumped into the coil using a syringe pump (approximate residence time of 60 min). At steady state, a good yield of **3e** was measured in the output stream.

**Fig. 4:**
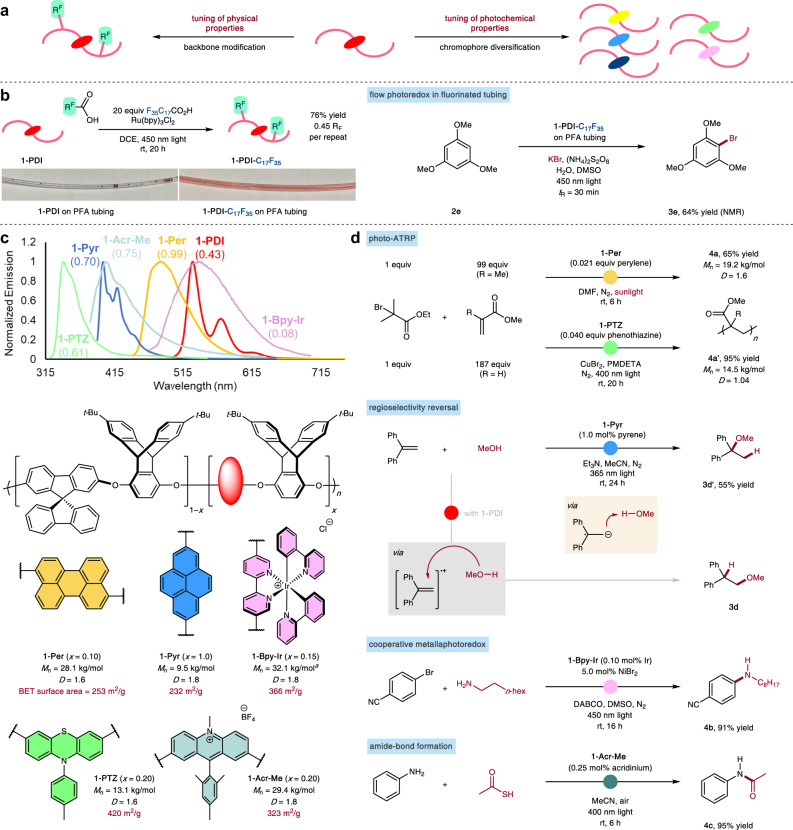
Tunability of the polymer catalyst. **a** Convenient and independent alteration of catalyst properties. **b** Post-polymerization functionalization of the backbone enables adhesion to fluorinated surfaces for photoredox catalysis. **c** Structural variation in the photocatalytic moiety: incorporation of common photoredox chromophores. Numbers in parentheses indicate photoluminescence quantum yields in dilute THF solution (1.0 × 10^–5^–5.0 × 10^–7^ M). **d** Example reactions using modified photocatalysts. ^a^Molecular weight distribution measured on **1-Bpy** prior to Ir incorporation. PMDETA = 1,1,4,7,7-pentamethyldiethylenetriamine.

A unique advantage of linear polymers as an immobilization platform is the ability to independently adjust the backbone-derived physical characteristics and the chromophore-derived photochemical attributes (Fig. 4a). Achieving adhesion to fluoropolymer surfaces was desirable since inert, flexible substances such as perfluoroalkoxy (PFA) plastics are the most common tubing materials for modern continuous-flow synthesis. Since most poly(arylene ether) polymers such as **1-PDI** adsorb poorly to fluorinated surfaces, and therefore do not form high-quality films, we wondered if we could enhance the attractive interaction through post-polymerization functionalization of the backbone. We subjected the polymer to C–H fluoroalkylation conditions with photogenerated perfluoroalkyl (*n*-C_17_F_35_) radicals to obtain **1-PDI-C**_**17**_**F**_**35**_ with relatively high fluorine content (F/C ratio = 1:4 by XPS). This new polymer was successfully deposited on narrow inner diameter (1/16 in) PFA tubing and effectively employed as a catalytic stationary phase in the flow synthesis of **3e** (Fig. 4b).

The identity of the covalently-included dye and its doping concentration could be adjusted arbitrarily with little effect on the porosity, solution-processability, and film-forming properties of the polymer. The substitution of other groups in lieu of PDI is synthetically straightforward and requires essentially no procedural modifications (Fig. 4c). Perylenes are highly reducing photocatalysts that have been used in applications such as light-activated atom-transfer radical polymerization (photo-ATRP) reactions^41^. Using perylene-containing **1-Per** as a catalyst, the polymerization of methyl methacrylate could be performed using sunlight as the energy source. With minimal optimization, the poly(methyl methacrylate) (PMMA, **4a**) product could be obtained with good molecular weight and polydispersity. Furthermore, the catalytic polymer was easily separated from the product polymer, as the former is strictly insoluble in acetone. The outcome of photo-ATRP can be greatly improved by changing the chromophore and adding a copper cocatalyst. Using phenothiazine **1-PTZ**, a controlled radical polymerization reaction of methyl acrylate was achieved, with polydispersity as low as 1.04. These optimized results compare favorably to those using state-of-the-art conjugated microporous phenothiazine catalysts^42^, which are also significantly more cumbersome to process and to characterize in terms of active site structure and density.

By altering the excited-state redox potentials of catalysts, the preferred mechanistic pathway of a reaction can be switched. Previous studies of the hydroetherification of **2d** have revealed that the use of different organic photocatalysts can engender divergent regiochemical outcomes^32^. Above, we showed that linear product is exclusively formed when using **1-PDI** as the photocatalyst. When, instead, the pyrene **1-Pyr** and long-wave UV (365 nm) irradiation were used, high branched selectivity was observed (**3d’**), attributable to the favorability of a photo-reductive, rather than oxidative, route.

In addition to the organic catalysts described above, organometallic dyes can also be easily incorporated through polymer post-functionalization. We were able to coordinate iridium to a porous bipyridine-based material (**1-Bpy-Ir**), producing chelated iridium complexes that resemble prototypical organometallic photocatalysts^7^. Iridium photocatalysts can initiate and maintain a Ni(I/III)-based catalytic cycle for Buchwald–Hartwig C–N cross-coupling^43,44^, a staple transformation in pharmaceutical research and development. Using a low loading of **1-Bpy-Ir** in conjunction with catalytic NiBr_2_, the amination of an aryl bromide was accomplished in a clean and high-yielding manner (product **4b**), without the requirement for any additional ligand for nickel. Finally, as cationic photocatalysts are frequently employed to access the most oxidizing excited-state species, we aimed to demonstrate that charged chromophores such as acridiniums could be incorporated through post-polymerization alkylation. The polymer **1-Acr-Me** was synthesized in high yield and found to be an extraordinarily active heterogeneous photocatalyst, promoting the direct acylation of aniline at nearly an order-of-magnitude reduced acridinium loading compared to previously reported homogenous reactions^45^.

Collectively, our findings imply that a broad scope of photocatalytic chromophores, from common to yet-undiscovered, could be integrated by copolymerization into the rigid structural framework of **1**. As the scaffold consistently bestows high specific surface area, excellent film properties, and processability in select non-polar solvents, the described method may constitute a general strategy for the covalent heterogenization of catalysts into porous materials for efficient and economical organic synthesis. In particular, we anticipate that these porous-polymer films, in the role of photocatalytic stationary phases in continuous flow, could represent an enabling technology for large-scale chemical manufacturing.

## Methods

### Example photocatalytic reaction: synthesis of methyl phenyl sulfoxide (3a)

A dry 20 mL scintillation vial, equipped with a magnetic stir bar, was charged sequentially with **1-PDI** (1.34 mg, 0.18 μmol, 0.018 mol% PDI), methanol (4.0 mL), and methyl phenyl sulfide (124.2 mg, 1.0 mmol, 1.0 equiv). The vial was submerged into an ultrasonic bath for 1 min to disperse the catalyst. The vial was capped with a screw cap containing a rubber septum, which was punctured with two 18-gauge needles to vent the reaction mixture to the atmosphere. The cloudy red suspension was stirred vigorously at rt under illumination from a 450 nm blue LED lamp for 6 h. At this point, both the irradiation and the stirring were stopped, and the catalyst allowed to settle for 30 min. Additional methanol (1.0 mL) was added, and the mixture was filtered through a short plug of Celite, washing with methanol (5.0 mL). The filtrate was concentrated with the aid of a rotary evaporator, and the title compound was obtained as a clear oil (133.1 mg, 95% yield) after column chromatography on silica gel, using DCM as the eluent. The catalytic polymer **1-PDI** could be recovered from the Celite by washing with THF (10 mL). ^**1**^**H NMR** (500 MHz, CDCl_3_) δ 7.67–7.61 (m, 2H), 7.55–7.47 (m, 3H), 2.72 (s, 3H). ^**13**^**C NMR** (125 MHz, CDCl_3_) δ 145.9, 131.1, 129.5, 123.6, 44.1. The spectral data agreed closely with those reported in the literature^46^.

## Supplementary information

Supporting Information

Description of Additional Supplementary Files

Movie 1

Movie 2

## Data Availability

Additional experimental methods and data are available in the Supplementary Information of this paper.
