# Supplementary material for: Solution-processable microporous polymer platform for heterogenization of diverse photoredox catalysts
Source: Nat Commun. 2022 May 27;13:2775. doi: 10.1038/s41467-022-29811-6 (PMC9142596; doi:10.1038/s41467-022-29811-6)
Supplement: Supplementary file 1 — Supporting Information [file 41467_2022_29811_MOESM1_ESM.docx]

Supplementary Information for

**Solution-Processable Microporous Polymer Platform for Heterogenization of Diverse Photoredox Catalysts**

Richard Y. Liu*^,a,b^, Sheng Guo*^,a,b^, Shao-Xiong Lennon Luo^a,b^, and Timothy M. Swager^a,b^

^a^Institute for Soldier Nanotechnologies, 500 Technology Square, Cambridge, MA 02139, USA

^b^Department of Chemistry, 77 Massachusetts Avenue, Cambridge, MA 02139, USA

# Contents

[**Supplementary Methods 3**](#_Toc96809177)

[1. General Information for Materials and Analytical Methods 3](#_Toc96809178)

[2. Synthesis of Polymers 6](#_Toc96809179)

[3. Characterization Data for Polymers 8](#_Toc96809180)

[3.1. Porous Heterogenous Organic Photocatalysts 8](#_Toc96809181)

[3.2. Porous Organometallic Photocatalyst 13](#_Toc96809182)

[3.3. Perfluoroalkylation of Porous Heterogenous Organic Photocatalysts 15](#_Toc96809183)

[4. NMR Spectra for Polymers 17](#_Toc96809184)

[5. Gel Permeation Chromatography 29](#_Toc96809185)

[6. N_2_ Isotherm and Pore-Size Distribution Data 33](#_Toc96809186)

[7. FT-IR Spectra 36](#_Toc96809187)

[8. X-Ray Photoelectron Spectroscopy 45](#_Toc96809188)

[9. Thermogravimetric Analysis and Differential Scanning Calorimetry 48](#_Toc96809189)

[10. Photophysical Data and Methods 51](#_Toc96809190)

[10.1. UV-Vis Absorbance Spectra 51](#_Toc96809191)

[10.2. Fluorescence Anisotropy 51](#_Toc96809192)

[10.3. Reversible Redox Activity 53](#_Toc96809193)

[10.4. Photoconductivity Measurements 53](#_Toc96809194)

[11. Synthetic Procedures for Photoredox Catalysis 56](#_Toc96809195)

[11.1. Experimental Procedure for Kinetic Studies 56](#_Toc96809196)

[11.2. Synthetic Examples 57](#_Toc96809197)

[11.3. Film Catalysis and Recycling 63](#_Toc96809198)

[11.4. Textile Catalysis 64](#_Toc96809199)

[11.5. Magnetic Particle Catalysis 65](#_Toc96809200)

[11.6. Continuous-Flow Chemistry 66](#_Toc96809201)

[12. NMR Spectra of Photoredox Products 70](#_Toc96809202)

[**Supplementary References 91**](#_Toc96809203)

# Supplementary Methods

# General Information for Materials and Analytical Methods

Unless noted, all chemicals were obtained from commercial sources, stored at room temperature, and used as received. All preparative C–O cross-coupling reactions were carried out under an atmosphere of nitrogen using standard Schlenk techniques. Anhydrous 2-methyltetrahydrofuran (2-MeTHF), toluene, and dimethoxyethane (DME) were purchased from Sigma-Aldrich in Sure-Seal^TM^ bottles and were degassed prior to use by sparging the liquid with nitrogen gas for 10 min while submerged in a sonication bath. *^t^*BuBrettPhos and other phosphine ligands, palladium(π-cinnamyl) chloride dimer, and potassium phosphate were purchased from Sigma-Aldrich. Other reagents were either prepared according to referenced literature procedures or were purchased from chemical suppliers (Sigma-Aldrich, Ambeed, TCI America, Combi-Blocks). Products were purified by flash chromatography using SiliCycle SiliaFlashP60 (230–400 mesh) silica gel with the aid of a Biotage Automated Flash Chromatography System.

Nuclear magnetic resonance (NMR): ^1^H, ^13^C, and ^19^F NMR spectra were recorded on a Bruker Avance-600 MHz or 500 MHz spectrometer. ^1^H and ^13^C spectra were calibrated using residual solvent as an internal reference (CHCl_3_: δ 7.26 ppm and δ 77.36 ppm, respectively; DMSO: 2.50 ppm and 39.52 ppm, respectively). Broadband ^1^H decoupling was used during the collection of ^13^C and ^19^F NMR spectra. The following abbreviations were used to denote multiplicities: s = singlet, bs = broad singlet, d = doublet, t = triplet, q = quartet, p = pentet, and m = multiplet.

Gel permeation chromatography (GPC): Measurements were carried out in HPLC-grade tetrahydrofuran using an Agilent 1260 Infinity system with variable wavelength diode array (254, 450m and 530 nm) and refractive index detectors, guard column (Agilent PLgel; 5 µm; 50 x 7.5 mm), and three analytical columns (Agilent PLgel; 5µm; 300 x 7.5 mm; 105, 104, and 103 Å pore sizes). The instrument was calibrated with narrow-dispersity polystyrene standards between 1.7 and 3150 kg mol^−1^. All runs were performed at 1.0 mL min^−1^ flow rate and 35 ºC. Molecular weight values were calculated using ChemStation GPC Data Analysis Software (Rev. B.01.01) based on the refractive index signal.

Thermogravimetric analysis (TGA): TGA measurements were carried out under nitrogen atmosphere (AirGas, ultra-high purity grade) using a TGA 550 from TA Instruments. The ramp speed was 10 °C min^−1^, and isotherms were performed from room temperature to 900 °C. The degradation temperature (T_d_) of a polymer is the temperature at which the polymer loses 10% of its initial mass. Thermal transitions were determined by differential scanning calorimetry (DSC) using a Discovery DSC from TA instruments with powdered samples (5-8 mg) sealed in aluminum pans.

Brunauer–Emmett–Teller (BET) measurements: BET surface areas of polymers were measured with N_2_ sorption at 77 K using a Micromeritics ASAP 2020. Analysis of pore-size distributions was performed using the Non-Local Density Functional Theory (NLDFT) model for carbon slit pore geometry provided by ASAP 2020.

X-ray photoelectron spectroscopy (XPS): XPS measurements were performed on a Thermo Scientific K-Alpha+ X-ray photoelectron spectrometer with a hemispherical energy analyzer and a monochromated X-ray source (Al K-alpha). Survey and high-resolution scans were collected with an X-ray spot size of 400 μm and a pass energy of 200 and 50 eV, respectively. XPS spectra were processed with the Avantage software.

Fourier-transform infrared (FT-IR) spectroscopy: All IR spectra were obtained on a Thermo Scientific Nicolet iS5 spectrometer (iD5 ATR, diamond).

Optical spectroscopy: Ultraviolet-visible (UV-Vis) absorption spectrometry was performed on an Agilent Cary 5000 instrument. Photoluminescence measurements were carried out using a HORIBA Jobin Yvon Fluorolog-3 spectrofluorometer (model FL-321). Solution quantum yields were measured relative to that of a known standard (fluorescein in 1.0 M NaOH) by taking UV-Vis and photoluminescence spectra over a range of dilute concentrations (1.0 × 10^–5^ to 5.0 × 10^–7^ M). Solid-state quantum yields were obtained on powders using an integrating sphere accessory.

Photoconductivity experiments: The resistance of film samples was measured using an Agilent Keysight 34970A galvanostat equipped with a 34901A 20-channel multiplexer (2/4-wire) module. The potentiostat was connected to a computer using an Agilent 82357B GPIBUSB interface high-speed USB 2.0 serial cable and controlled using BenchLink Data Logger 3.

High-resolution mass spectrometry (HRMS): ESI-HRMS (electrospray ionization) and DART-HRMS (direct analysis in real time) spectrometric data were recorded on a Bruker Daltonics APEXIV 4.7 Tesla Fourier-transform ion cyclotron resonance mass spectrometer (FT-ICR-MS). DART-MS spectrometric data were also recorded on an IonSence Inc. DART SVP mass spectrometer.

Residual metal analysis: For sample preparation, a 20 mL vial was charged with polymer (10 mg), concentrated nitric acid (3.0 mL, 70%), and an egg-shaped magnetic stir bar. The vial was capped and allowed to stir at rt overnight. The mixture was carefully diluted with water (10 mL). After filtration, the aqueous solution was further diluted using a 100 mL volumetric flask. The Pd content of this solution was determined using inductively-coupled plasma mass spectrometry (ICP-MS).

Heavy-wall cylindrical vessels were purchased from Synthware (P260001). These vessels have an internal thread for use with a 15 mm PTFE bushing as a pressure seal and a special 0–4 mm vacuum valve on a side tube (Supplementary Supplementary Figure 1).


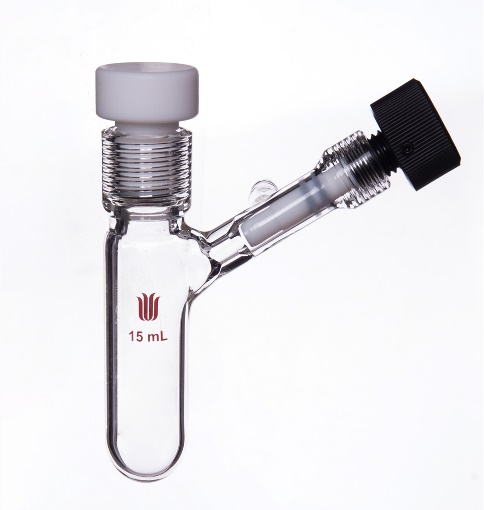


**Supplementary Figure 1.** Pressure vessel (P260001).

# Synthesis of Polymers

***General Procedure*:** An oven-dried pressure vessel (P260001 heavy wall, with side port, 15 mL), equipped with a stir bar, was sequentially charged with the solid reagents, palladium(π-cinnamyl) chloride dimer (2.6 mg, 0.005 mmol, 0.01 equiv), *^t^*BuBrettPhos (15 mg, 0.03 mmol, 0.06 equiv), *^t^*Bu-triptycene hydroquinone (199 mg, 0.5 mmol, 1.0 equiv), 2,7-dibromo-9,9'-spirobifluorene (237 mg, 0.5 mmol, 1.0 equiv), and K_3_PO_4_ (318 mg, 1.5 mmol, 3.0 equiv). The reaction tube was evacuated and backfilled with nitrogen from the Schlenk line (this process was repeated a total of five times), after which toluene (0.6 mL) and dimethoxyethane (0.3 mL) were added successively. The reaction tube was placed into a preheated oil bath, and the mixture was stirred at 140 °C for 20 h.

***Workup I***: The reaction tube was removed from the oil bath and allowed to cool to rt. Next, the tube was uncapped, and the reaction solution was diluted with THF (5.0 mL) and aqueous HCl (1.0 M, 3.0 mL). The crude material was transferred to a separatory funnel, and more THF (5.0 mL) and brine (10 mL) were added. The aqueous phase was removed, and the organic phase was washed once with brine (5.0 mL). The solution containing the crude product was dried over with Na_2_SO_4_ and concentrated to ~5 mL with the aid of a rotary evaporator. Upon completion, the resulting polymer was precipitated by adding methanol (50 mL). The precipitation process was repeated by redissolving the solid in THF and adding methanol again. The collected polymer was dried in a vacuum oven at 140 °C overnight before characterization.

***Workup II***: The reaction tube was removed from the oil bath and allowed to cool to rt. Next, the tube was uncapped, and the reaction solution was diluted with THF (5.0 mL) and 0.2 M thiourea aqueous solution (5 mL). The mixture was allowed to stir at rt for 4 h. The crude material was transferred to a separatory funnel, and more THF (5.0 mL) and brine (10 mL) were added. The aqueous phase was removed, and the organic phase was washed once with brine (5.0 mL). The solution containing the crude product was dried over with Na_2_SO_4_ and concentrated to ~5 mL with the aid of a rotary evaporator. Upon completion, the resulting polymer was precipitated by adding methanol (50 mL). The precipitation process was repeated by redissolving the solid in THF and adding methanol again. The collected polymer was dried in a vacuum oven at 140 °C overnight before characterization.

# Characterization Data for Polymers

## 3.1. Porous Heterogenous Organic Photocatalysts

**1-PDI:** The general procedure was followed on a 0.5 mmol scale using palladium(π-cinnamyl) chloride dimer (2.6 mg, 5.0 µmol, 1.0 mol%), *^t^*BuBrettPhos (15 mg, 0.03 mmol, 0.06 equiv), 6,15-di-*tert*-butyl-9,10-dihydro-9,10-[1,2]benzenoanthracene-1,4-diol^1,2^ (199 mg, 0.5 mmol, 1.0 equiv), 2,7-dibromo-9,9'-spirobifluorene (213 mg, 0.45 mmol, 0.9 equiv), and 5,12-dibromo-2,9-bis(2-ethylhexyl)anthra[2,1,9-def:6,5,10-d'e'f']diisoquinoline-1,3,8,10(2*H*,9*H*)-tetraone (40 mg, 0.05 mmol, 0.1 equiv). The reaction mixture was stirred at 140 °C for 20 h. After ***Workup I*** , the title compound was obtained as a red powder (350 mg, 95% yield). **^1^H NMR** (600 MHz, CDCl_3_) *SBF-TBTrip part*: δ 7.92 – 7.85 (m, 2H), 7.51 (t, *J* = 7.8 Hz, 2H), 7.47 – 7.39 (m, 2H), 7.23 – 7.15 (m, 4H), 7.10 (dd, *J* = 7.8, 4.1 Hz, 2H), 6.99 – 6.88 (m, 4H), 6.62 (d, *J* = 8.2 Hz, 2H), 6.54 (dd, *J* = 18.3, 8.2 Hz, 2H), 6.43 (t, *J* = 6.2 Hz, 2H), 5.52 (d, *J* = 17.7 Hz, 2H), 1.22 (s, 18H); *PDI part*: δ 9.95 – 9.65 (m, 0.2 H), 8.94 – 8.59 (m, 0.4H), 4.43 – 3.77 (m, 0.4H). **IR** (neat, cm^−1^) 3067, 2961, 2868, 1700, 1662, 1595, 1478, 1459, 1436, 1363, 1261, 1221, 1130, 1107, 996, 909, 866, 812, 736, 637. Molecular weight values were calculated using ChemStation GPC Data Analysis Software (Rev. B.01.01) based on the refractive index signal. *M*n = 15.7 kg/mol, *M*w = 24.0 kg/mol, PDI = 1.5 (Supplementary Figure 2-1). The porosity of the polymer powder was measured using nitrogen adsorption isotherm at 77 K with a saturation pressure of P_0_ = 1 bar. BET surface area was calculated based on a P/P_0_ range from 0.06 to 0.20 for the adsorption measurement. **1-PDI** has a Brunauer–Emmett–Teller (BET) internal surface area of 338 m^2^g^−1^. Pore-size distributions were analyzed using NLDFT based on the adsorption part of isotherm and carbon slit pore geometry (Supplementary Figure 3-1). ICP-MS analysis of the polymer product showed that the amount of Pd residue in the polymer was 38 ppm**.**

**1-PDI-HQ:** The general procedure was followed on a 0.5 mmol scale using palladium(π-cinnamyl) chloride dimer (2.6 mg, 5.0 µmol, 1.0 mol%), *^t^*BuBrettPhos (15 mg, 0.03 mmol, 0.06 equiv), hydroquinone (55 mg, 0.5 mmol, 1.0 equiv), 2,7-dibromo-9,9'-spirobifluorene (213 mg, 0.45 mmol, 0.9 equiv), and 5,12-dibromo-2,9-bis(2-ethylhexyl)anthra[2,1,9-def:6,5,10-d'e'f']diisoquinoline-1,3,8,10(2*H*,9*H*)-tetraone (40 mg, 0.05 mmol, 0.1 equiv). The reaction mixture was stirred at 140 °C for 20 h. After ***Workup I***, the title compound was obtained as a red powder (218 mg, 96% yield). **^1^H NMR** (600 MHz, CDCl_3_) *SBF-HQ part*: δ 7.78 (d, *J* = 7.7 Hz, 2H), 7.64 (d, *J* = 8.4 Hz, 2H), 7.34 (s, 2H), 7.26 (s, 2H), 7.11 (s, 2H), 6.86 (d, *J* = 8.6 Hz, 2H), 6.82 – 6.68 (m, 6H), 6.39 (s, 2H); *PDI part*: δ 9.56 – 9.43 (m, 0.2H), 8.71 – 8.53 (m, 0.4H), 4.37 – 4.01 (m, 0.4H), 2.01 – 1.79 (m, 0.2H), 1.62 – 1.08 (m, 1.6H), 1.07 – 0.76 (m, 1.2H). **IR** (neat, cm^−1^) 3064, 3041, 2958, 2870, 1698, 1658, 1596, 1496, 1460, 1435, 1256, 1208, 1193, 1106, 985, 909, 869, 843, 817, 739, 637, 620. Molecular weight values were calculated using ChemStation GPC Data Analysis Software (Rev. B.01.01) based on the refractive index signal. *M*n = 23.9 kg/mol, *M*w = 38.6 kg/mol, PDI = 1.6. (Supplementary Figure 2-2) The porosity of the polymer powder was measured using nitrogen adsorption isotherm at 77 K with a saturation pressure of P_0_ = 1 bar. Brunauer–Emmett–Teller (BET) analysis for **1-PDI-HQ** was unable to give meaningful data since the surface area is very low.

**1-Per:** The general procedure was followed on a 0.5 mmol scale using palladium(π-cinnamyl) chloride dimer (2.6 mg, 5.0 µmol, 1.0 mol%), *^t^*BuBrettPhos (15 mg, 0.03 mmol, 0.06 equiv), 6,15-di-*tert*-butyl-9,10-dihydro-9,10-[1,2]benzenoanthracene-1,4-diol (199 mg, 0.5 mmol, 1.0 equiv), 2,7-dibromo-9,9'-spirobifluorene (213 mg, 0.45 mmol, 0.9 equiv), and 3,9-dibromoperylene (21 mg, 0.05 mmol, 0.1 equiv). The reaction mixture was stirred at 140 °C for 20 h. After ***Workup I*** , the title compound was obtained as a yellow powder (330 mg, 94% yield). **^1^H NMR** (600 MHz, CDCl_3_) *SBF-TBTrip part*: δ 7.92 – 7.85 (m, 2H), 7.51 (t, *J* = 7.8 Hz, 2H), 7.47 – 7.39 (m, 2H), 7.23 – 7.15 (m, 4H), 7.10 (dd, *J* = 7.8, 4.1 Hz, 2H), 6.99 – 6.88 (m, 4H), 6.62 (d, *J* = 8.2 Hz, 2H), 6.54 (dd, *J* = 18.3, 8.2 Hz, 2H), 6.43 (t, *J* = 6.2 Hz, 2H), 5.52 (d, *J* = 17.7 Hz, 2H), 1.22 (s, 18H); *Perylene part:* δ 8.40 – 8.30 (m, 0.4H). **IR** (neat, cm^−1^) 3064, 3037, 2960, 2868, 1606, 1583, 1477, 1458, 1435, 1260, 1219, 1130, 1107, 1016, 996, 909, 865, 810, 735, 637, 620. Molecular weight values were calculated using ChemStation GPC Data Analysis Software (Rev. B.01.01) based on the refractive index signal. *M*n = 28.1 kg/mol, *M*w = 45.0 kg/mol, PDI = 1.6 (Supplementary Figure 2-3). The porosity of the polymer powder was measured using nitrogen adsorption isotherm at 77 K with a saturation pressure of P_0_ = 1 bar. BET surface area was calculated based on a P/P_0_ range from 0.06 to 0.20 for the adsorption measurement. **1-PDI-Per** has a Brunauer–Emmett–Teller (BET) internal surface area of 253 m^2^g^−1^. Pore-size distributions were analyzed using NLDFT based on the adsorption part of isotherm and carbon slit pore geometry (Supplementary Figure 3-2). ICP-MS analysis of the polymer product showed that the amount of Pd residue in the polymer was 33 ppm.

**1-Pyr:** The general procedure was followed on a 0.5 mmol scale using palladium(π-cinnamyl) chloride dimer (2.6 mg, 5.0 µmol, 1.0 mol%), *^t^*BuBrettPhos (15 mg, 0.03 mmol, 0.06 equiv), 6,15-di-*tert*-butyl-9,10-dihydro-9,10-[1,2]benzenoanthracene-1,4-diol (199 mg, 0.5 mmol, 1.0 equiv), and 2,7-dibromopyrene (95% purity, 189 mg, 0.5 mmol, 1.0 equiv). The reaction mixture was stirred at 140 °C for 20 h. After ***Workup I***, the title compound was obtained as a gray powder (275 mg, 92% yield). **^1^H NMR** (600 MHz, CDCl_3_) δ 8.00 – 7.90 (m, 4H), 7.83 – 7.75 (m, 4H), 7.27 – 7.21 (m, 2H), 7.17 – 7.10 (m, 2H), 7.07 – 6.96 (m, 2H), 6.88 (s, 2H), 5.86 – 5.76 (m, 2H), 1.21 (s, 18H). **IR** (neat, cm^−1^) 3046, 2959, 2903, 2887, 1595, 1476, 1437, 1362, 1279, 1264, 1217, 1136, 1017, 995, 968, 906, 866, 826, 808, 729, 704, 648. Molecular weight values were calculated using ChemStation GPC Data Analysis Software (Rev. B.01.01) based on the refractive index signal. *M*n = 9.5 kg/mol, *M*w = 17.1 kg/mol, PDI = 1.8 (Supplementary Figure 2-4). The porosity of the polymer powder was measured using nitrogen adsorption isotherm at 77 K with a saturation pressure of P_0_ = 1 bar. BET surface area was calculated based on a P/P_0_ range from 0.05 to 0.20 for the adsorption measurement. **1-Pyr** has a Brunauer–Emmett–Teller (BET) internal surface area of 232 m^2^g^−1^. Pore-size distributions were analyzed using NLDFT based on the adsorption part of isotherm and carbon slit pore geometry (Supplementary Figure 3-3). ICP-MS analysis of the polymer product showed that the amount of Pd residue in the polymer was 30 pm.

**1-PTZ:** The general procedure was followed on a 0.5 mmol scale using palladium(π-cinnamyl) chloride dimer (2.6 mg, 5.0 µmol, 1.0 mol%), *^t^*BuBrettPhos (15 mg, 0.03 mmol, 0.06 equiv), 6,15-di-*tert*-butyl-9,10-dihydro-9,10-[1,2]benzenoanthracene-1,4-diol (199 mg, 0.5 mmol, 1.0 equiv), 2,7-dibromo-9,9'-spirobifluorene (190 mg, 0.40 mmol, 0.8 equiv), and 3,7-dibromo-10-(*p*-tolyl)-10*H*-phenothiazine (45 mg, 0.1 mmol, 0.2 equiv).3 The reaction mixture was stirred at 140 °C for 20 h. After ***Workup II***, the title compound was obtained as a gray powder (340 mg, 96% yield). **^1^H NMR** (600 MHz, CDCl_3_) *SBF-TBTrip part*: δ 7.92 – 7.85 (m, 2H), 7.51 (t, *J* = 7.8 Hz, 2H), 7.47 – 7.39 (m, 2H), 7.23 – 7.15 (m, 4H), 7.10 (dd, *J* = 7.8, 4.1 Hz, 2H), 6.99 – 6.88 (m, 4H), 6.62 (d, *J* = 8.2 Hz, 2H), 6.54 (dd, *J* = 18.3, 8.2 Hz, 2H), 6.43 (t, *J* = 6.2 Hz, 2H), 5.52 (d, *J* = 17.7 Hz, 2H), 1.22 (s, 18H); *N-(p-tolyl)-10H-phenothiazine part:* δ 6.30 – 6.20 (m, 0.4H), 6.18 – 6.08 (m, 0.4H), 2.46 – 2.40 (m, 0.6H). **IR** (neat, cm^−1^) 3064, 2962, 2904, 2869, 1605, 1582, 1477, 1459, 1436, 1363, 1260, 1220, 1130, 1106, 1016, 996, 924, 865, 825, 811, 743, 637, 620, 493, 438, 417. Molecular weight values were calculated using ChemStation GPC Data Analysis Software (Rev. B.01.01) based on the refractive index signal. *M*n = 13.1 kg/mol, *M*w = 20.4 kg/mol, PDI = 1.6 (Supplementary Figure 2-5). The porosity of the polymer powder was measured using nitrogen adsorption isotherm at 77 K with a saturation pressure of P_0_ = 1 bar. BET surface area was calculated based on a P/P_0_ range from 0.06 to 0.20 for the adsorption measurement. **1-PTZ** has a Brunauer–Emmett–Teller (BET) internal surface area of 420 m^2^g^−1^. Pore-size distributions were analyzed using NLDFT based on the adsorption part of isotherm and carbon slit pore geometry (Supplementary Figure 3-5). ICP-MS analysis of the polymer product showed that the amount of Pd residue in the polymer was 86 ppm.

**1-Acr:** The general procedure was followed on a 1.0 mmol scale using palladium(π-cinnamyl) chloride dimer (5.2 mg, 10.0 µmol, 1.0 mol%), *^t^*BuBrettPhos (30 mg, 0.06 mmol, 0.06 equiv), 6,15-di-*tert*-butyl-9,10-dihydro-9,10-[1,2]benzenoanthracene-1,4-diol (399 mg, 1.0 mmol, 1.0 equiv), 2,7-dibromo-9,9'-spirobifluorene (380 mg, 0.80 mmol, 0.8 equiv), and 2,7-dibromo-9-mesitylacridine (91 mg, 0.2 mmol, 0.2 equiv).^4^ The reaction mixture was stirred at 140 °C for 42 h. After ***Workup II***, the title compound was obtained as a gray powder (700 mg, 98% yield). **^1^H NMR** (600 MHz, CDCl_3_) *SBF-TBTrip part*: δ 7.92 – 7.85 (m, 2H), 7.51 (t, *J* = 7.8 Hz, 2H), 7.47 – 7.39 (m, 2H), 7.23 – 7.15 (m, 4H), 7.10 (dd, *J* = 7.8, 4.1 Hz, 2H), 6.99 – 6.88 (m, 4H), 6.62 (d, *J* = 8.2 Hz, 2H), 6.54 (dd, *J* = 18.3, 8.2 Hz, 2H), 6.43 (t, *J* = 6.2 Hz, 2H), 5.52 (d, *J* = 17.7 Hz, 2H), 1.22 (s, 18H); *9-mesitylacridine part:* δ 8.25 – 8.13 (m, 0.4H), 2.43 – 2.34 (m, 0.6H), 1.78 – 1.70 (m, 1.2H). Molecular weight values were calculated using ChemStation GPC Data Analysis Software (Rev. B.01.01) based on the refractive index signal. *M*n = 29.5 kg/mol, *M*w = 54.3 kg/mol, PDI = 1.9 (Supplementary Figure 2-6).

**1-Acr-Me**: An oven-dried screw-cap reaction tube (20 mm × 125 mm, Fisher part #1495937A), equipped with a stir bar, was sequentially charged with **1-Acr** (71 mg, 0.1 mmol, 1 equiv) and trimethyloxonium tetrafluoroborate (60 mg, 0.4 mmol, 4 equiv). The reaction tube was evacuated and backfilled with nitrogen from the Schlenk line (this process was repeated a total of five times), after which chloroform (0.5 mL) and DIPEA (13 mg, 0.1 mmol, 1 equiv) were added. The mixture was stirred at 65 ^°^C for 20 h. Upon completion, the resulting polymer was precipitated by adding the crude reaction mixture to anhydrous ether (50 mL). The collected polymer was washed with methanol (20 mL) and dried in a vacuum oven at 80 °C overnight before characterization. **1-Acr-Me** was obtained as a yellow powder (80 mg, 96% yield). **^1^H NMR** (600 MHz, CDCl_3_) *SBF-TBTrip part*: δ 7.92 – 7.85 (m, 2H), 7.51 (t, *J* = 7.8 Hz, 2H), 7.47 – 7.39 (m, 2H), 7.23 – 7.15 (m, 4H), 7.10 (dd, *J* = 7.8, 4.1 Hz, 2H), 6.99 – 6.88 (m, 4H), 6.62 (d, *J* = 8.2 Hz, 2H), 6.54 (dd, *J* = 18.3, 8.2 Hz, 2H), 6.43 (t, *J* = 6.2 Hz, 2H), 5.52 (d, *J* = 17.7 Hz, 2H), 1.21 (s, 18H); *Acr-MeBF_4_* *part:* δ 8.80 – 8.61 (m, 0.4H), 5.07 (s, 0.6H), 2.43 – 2.22 (m, 0.6H), 1.73 – 1.54 (m, 1.2H). **^19^F NMR** (471 MHz, CDCl_3_): δ -153.0. **IR** (neat, cm^−1^) 3066, 2962, 2904, 2869, 1609, 1582, 1553, 1477, 1458, 1436, 1415, 1393, 1363, 1260, 1219, 1130, 1107, 1060, 1016, 996, 924, 865, 825, 744, 637, 620, 509, 492, 452, 418. The porosity of the polymer powder was measured using nitrogen adsorption isotherm at 77 K with a saturation pressure of P_0_ = 1 bar. BET surface area was calculated based on a P/P_0_ range from 0.05 to 0.20 for the adsorption measurement. **1-Acr-Me** has a Brunauer–Emmett–Teller (BET) internal surface area of 322 m^2^g^−1^. Pore-size distributions were analyzed using NLDFT based on the adsorption part of isotherm and carbon slit pore geometry (Supplementary Figure 3-6). ICP-MS analysis of the polymer product showed that the amount of Pd residue in the polymer was 30 ppm.

## 3.2. Porous Organometallic Photocatalyst

**1-Bpy:** The general procedure was followed on a 1.0 mmol scale using palladium(π-cinnamyl) chloride dimer (5.2 mg, 10.0 µmol, 1.0 mol%), *^t^*BuBrettPhos (30 mg, 0.06 mmol, 0.06 equiv), 6,15-di-*tert*-butyl-9,10-dihydro-9,10-[1,2]benzenoanthracene-1,4-diol (399 mg, 1.0 mmol, 1.0 equiv), 5,5'-dibromo-2,2'-bipyridine (48 mg, 0.15 mmol, 0.15 equiv), and 2,2’-dibromo-9,9'-spirobifluorene (403 mg, 0.85 mmol, 0.85 equiv). The reaction mixture was stirred at 140 °C for 48 h. After ***Workup II***, the title compound was obtained as a gray powder (650 mg, 95% yield). **^1^H NMR** (500 MHz, CDCl_3_) *SBF-TBTrip part*: δ 7.92 – 7.85 (m, 2H), 7.51 (t, *J* = 7.8 Hz, 2H), 7.47 – 7.39 (m, 2H), 7.23 – 7.15 (m, 4H), 7.10 (dd, *J* = 7.8, 4.1 Hz, 2H), 6.99 – 6.88 (m, 4H), 6.62 (d, *J* = 8.2 Hz, 2H), 6.54 (dd, *J* = 18.3, 8.2 Hz, 2H), 6.43 (t, *J* = 6.2 Hz, 2H), 5.52 (d, *J* = 17.7 Hz, 2H), 1.21 (s, 18H); *Bipyridine part*: 8.50 – 8.39 (m, 0.3 H), 8.37 – 8.25 (m, 0.3 H). **IR** (neat, cm^−1^) 3066, 3039, 2961, 2868, 1606, 1582, 1477, 1457, 1436, 1362, 1260, 1219, 1141, 1130, 1107, 996, 909, 865, 828, 734, 637, 620. Molecular weight values were calculated using ChemStation GPC Data Analysis Software (Rev. B.01.01) based on the refractive index signal. *M*n = 32.1 kg/mol, *M*w = 58.9 kg/mol, PDI = 1.8 (Supplementary Figure 2-7).

**1-Bpy-Ir**: An oven-dried screw-cap reaction tube (20 mm × 125 mm, Fisher part #1495937A), equipped with a stir bar, was sequentially charged with **1-Bpy** (137 mg, 0.2 mmol, 1 equiv) and dichlorotetrakis(2-(2-pyridinyl)phenyl)diiridium(III) (16.1 mg, 0.015 mmol, 1 equiv). The reaction tube was evacuated and backfilled with nitrogen from the Schlenk line (this process was repeated a total of five times), after which chloroform (1.0 mL) was added. The mixture was stirred at rt for 20 h. Upon completion, the resulting polymer was precipitated by addition of the crude reaction mixture into ether/hexane (1:1, 50 mL total). The collected polymer was dried in a vacuum oven at 80 °C overnight before characterization. **1-Bpy-Ir** was obtained as a yellow powder (153 mg, 100% yield). **^1^H NMR** (600 MHz, CDCl_3_) *SBF-TBTrip part*: δ 7.92 – 7.85 (m, 2H), 7.51 (t, *J* = 7.8 Hz, 2H), 7.47 – 7.39 (m, 2H), 7.23 – 7.15 (m, 4H), 7.10 (dd, *J* = 7.8, 4.1 Hz, 2H), 6.99 – 6.88 (m, 4H), 6.62 (d, *J* = 8.2 Hz, 2H), 6.54 (dd, *J* = 18.3, 8.2 Hz, 2H), 6.43 (t, *J* = 6.2 Hz, 2H), 5.52 (d, *J* = 17.7 Hz, 2H), 1.21 (s, 18H); *Ir complex part:* δ 9.65 – 9.28 (m), 7.83 – 7.72 (m), 6.84 – 6.70 (m). **IR** (neat, cm^−1^) 3064, 3039, 2962, 1607, 1583, 1477, 1459, 1436, 1363, 1261, 1220, 1130, 1107, 995, 909, 865, 827, 737, 637, 620. The porosity of the polymer powder was measured using nitrogen adsorption isotherm at 77 K with a saturation pressure of P_0_ = 1 bar. BET surface area was calculated based on a P/P_0_ range from 0.05 to 0.20 for the adsorption measurement. **1-Bpy-Ir** has a Brunauer–Emmett–Teller (BET) internal surface area of 366 m^2^g^−1^. Pore-size distributions were analyzed using NLDFT based on the adsorption part of isotherm and carbon slit pore geometry (Supplementary Figure 3-4). ICP-MS analysis of the polymer product showed that the amount of Pd residue in the polymer was 10 ppm.

## 3.3. Perfluoroalkylation of Porous Heterogenous Organic Photocatalysts

The procedure is adapted from a literature report.^5^ An oven-dried Schlenk tube (30 mL), equipped with a stir bar, was sequentially charged with the solid reagents, Ru(bpy)_3_Cl_2_·6H_2_O (3.8 mg, 0.005 mmol, 0.01 equiv), **1-PDI** (75 mg, 0.10 mmol, 1.0 equiv), perfluorooctadecanoic acid (1.82 g, 2.0 mmol, 20.0 equiv). The reaction tube was evacuated and backfilled with nitrogen from the Schlenk line (this process was repeated a total of five times), after which dichloroethane (10 mL) was added. The reaction mixture was degassed by sparging with nitrogen for 10 min with an outlet needle, then irradiated with 450 nm light by placing the flask ~15 cm from a 30 W LED flood light (Ustellar IP66 400 nm). After 24 h, the reaction mixture was neutralized with NaOH solution (2 M, 10 mL) and extracted with CH_2_Cl_2_ (3×30 mL). The combined organic phase was wash with brine, dried over with Na_2_SO_4_ and concentrated to ~5 mL with the aid of a rotary evaporator. The resulting polymer was precipitated by adding methanol (50 mL). The collected polymer was dried in a vacuum oven at 140 °C overnight. The perfluoroalkylated **1-PDI** was obtained as a red powder (83 mg, 76% yield). **^1^H NMR** (600 MHz, CDCl_3_): *SBF-TBTrip part*: δ 7.92 – 7.85 (m, 2H), 7.51 (t, *J* = 7.8 Hz, 2H), 7.47 – 7.39 (m, 2H), 7.23 – 7.15 (m, 4H), 7.10 (dd, *J* = 7.8, 4.1 Hz, 2H), 6.99 – 6.88 (m, 4H), 6.62 (d, *J* = 8.2 Hz, 2H), 6.54 (dd, *J* = 18.3, 8.2 Hz, 2H), 6.43 (t, *J* = 6.2 Hz, 2H), 5.52 (d, *J* = 17.7 Hz, 2H), 1.22 (s, 18H); *PDI part*: δ 9.95 – 9.65 (m, 0.2 H), 8.94 – 8.59 (m, 0.4H), 4.43 – 3.77 (m, 0.4H). **^19^F NMR** (471 MHz, CDCl_3_): δ -80.7, -121.6. **IR** (neat, cm^−1^) 3064, 3040, 2961, 1701, 1664, 1583, 1477, 1458, 1363, 1260, 1219, 1130, 1106, 995, 924, 888, 865, 811, 744, 637, 620, 493, 417. Based on X-ray photoelectron spectroscopy (XPS), the ratio of C and F of the polymer is 80:20. This ratio indicated that on average, 0.45 C_17_F_35_ units were incorporated on each repeat unit of perfluoroalkylated **1-PDI**. (Supplementary Figure 5-6). We note that perfluoroalkylation reactions using carboxylic acids as the radical source typically require the addition of an external terminal oxidant such as diphenyl sulfoxide. We found, however, that in the functionalization of 1-PDI, the reaction was equally effective with and without added sulfoxide. We wish to advise the reader that, in extending this functionalization protocol to other porous polymers not containing PDI units, the addition of sulfoxide may be necessary or beneficial and should be evaluated.

# NMR Spectra for Polymers

**^1^H NMR** (600 MHz, CDCl_3_)

**^1^H NMR** (600 MHz, CDCl_3_)

**^1^H NMR** (600 MHz, CDCl_3_)

**^1^H NMR** (600 MHz, CDCl_3_)

**^1^H NMR** (500 MHz, CDCl_3_)

**^1^H NMR** (500 MHz, CDCl_3_)

**^1^H NMR** (500 MHz, CDCl_3_)

**^19^F NMR** (471 MHz, CDCl_3_)

**^1^H NMR** (600 MHz, CDCl_3_)

**^1^H NMR** (600 MHz, CDCl_3_)

**^1^H NMR** (500 MHz, CDCl_3_)

**^19^F NMR** (471 MHz, CDCl_3_)

# Gel Permeation Chromatography

**Supplementary** **Figure 2-1.** GPC trace of **1-PDI.**

**Supplementary Figure 2-2.** GPC trace of **1-PDI-HQ.**

**Supplementary Figure 2-3.** GPC trace of **1-Per.**

**Supplementary Figure 2-4.** GPC trace of **1-Pyr**.

**Supplementary Figure 2-5.** GPC trace of **1-PTZ**.

**Supplementary Figure 2-6.** GPC trace of **1-Acr**.

**Supplementary Figure 2-7.** GPC trace of **1-Bpy**.

**Supplementary Figure 2-8.** GPC trace of controlled polymerization product **4a’**.

# N_2_ Isotherm and Pore-Size Distribution Data

**Supplementary Figure 3-1.** N_2_ adsorption-desorption isotherm and pore-size distribution of **1-PDI**.

**Supplementary Figure 3-2.** N_2_ adsorption–desorption isotherm and pore-size distribution of **1-Per**.

**Supplementary Figure 3-3.** N_2_ adsorption-desorption isotherm and pore-size distribution of **1-Pyr**.

**Supplementary Figure 3-4.** N_2_ adsorption-desorption isotherm and pore-size distribution of **1-Bpy-Ir**.

**Supplementary Figure 3-5.** N_2_ adsorption-desorption isotherm and pore-size distribution of **1-PTZ**.

**Supplementary Figure 3-6.** N_2_ adsorption-desorption isotherm and pore-size distribution of **1-Acr-Me**.

# FT-IR Spectra

**Supplementary Figure 4-1.** FT-IR spectrum of **1-PDI**.

**Supplementary Figure 4-2.** FT-IR spectra of **1-PDI** after exposure to different conditions for 24 hours. **Red**: 5 mg polymer, 5 mL boiling water, 24 h; **Blue**: 5 mg polymer, 5 mL 8 M NaOH, rt, 24 h; **Green**: 5 mg polymer, 5 mL 30% H_2_O_2_, rt, 24 h; **Dark Green**: 5 mg polymer, 5 mL 12 M HCl.

**Supplementary Figure 4-3.** FT-IR spectrum of **1-PDI-HQ**.

**Supplementary Figure 4-4.** FT-IR spectrum of **1-Per.**

**Supplementary Figure 4-5.** FT-IR spectrum of **1-Pyr**.

**Supplementary Figure 4-6.** FT-IR spectrum of **1-PTZ**.

**Supplementary Figure 4-7.** FT-IR spectrum of **1-Acr-Me**.

**Supplementary Figure 4-8.** FT-IR spectrum of **1-Bpy-Ir**.

**Supplementary Figure 4-9.** FT-IR spectrum of perfluoroalkylated **1-PDI**.

# X-Ray Photoelectron Spectroscopy


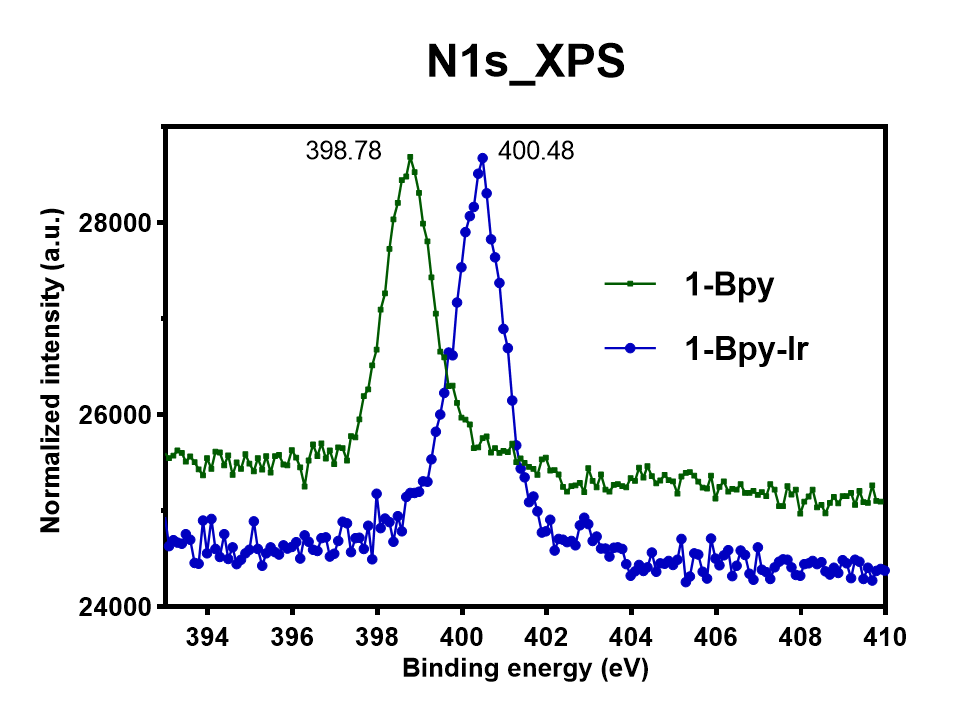


**Supplementary Figure 5-1.** N1*s* XPS spectra of **1-Bpy** and **1-Bpy-Ir**.

**Supplementary Figure 5-2.** Ir4*f* XPS spectrum of **1-Bpy-Ir**.

**Supplementary Figure 5-3.** XPS spectrum of **1-Bpy-Ir**. Atomic ratio (N:Ir) = 1 : 0.24.

**Supplementary Figure 5-4.** C1*s* XPS spectrum of perfluoroalkylated **1-PDI**.

**Supplementary Figure 5-5.** F1*s* XPS spectrum of perfluoroalkylated **1-PDI**.

**Supplementary Figure 5-6.** XPS spectrum of perfluoroalkylated **1-PDI**. Atomic ratio (C:F) = 80 : 20.

# Thermogravimetric Analysis and Differential Scanning Calorimetry

**Supplementary Figure 6-1.** TGA and DSC curves of **1-PDI**.

**Supplementary Figure 6-2.** TGA and DSC curves of **1-Per**.

**Supplementary Figure 6-3.** TGA and DSC curves of **1-Pyr**.

**Supplementary Figure 6-4.** TGA and DSC curves of **1-Bpy-Ir**.

**Supplementary Figure 6-5.** TGA curve of **1-PTZ**.

**Supplementary Figure 6-6.** TGA curve of **1-Acr-Me**.

**Supplementary Figure 6-7.** TGA curve of perfluoroalkylated **1-PDI**.

# Photophysical Data and Methods

## 10.1. UV-Vis Absorbance Spectra

**Supplementary Figure 7-1.** Absorbance measurements for catalytic polymers.

## 10.2. Fluorescence Anisotropy

The steady-state fluorescence polarization measurements were performed on thin films of **1-PDI** cast on glass slides by evaporation from THF solution. Two samples were prepared with similar optical densities as evaluated by UV-Vis absorbance spectroscopy: the first using pristine **1-PDI**, and the second using **1-PDI** with added quencher (*N*,*N*-dimethylaniline) at a sufficient quantity to reduce the photoluminescence roughly threefold. The measurement of polarization was carried out in a right-angle setup with adjustable polarizers in both the excitation and emission paths. The anisotropy value *r* is defined as:


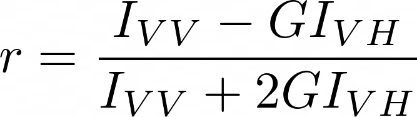


where *I* refers to intensity, with the first subscript indicating vertical (V) or horizontal (H) polarization at the excitation beam and the second indicating polarization at the emission. *G* refers to a wavelength-dependent grating factor that was determined by scattering from a colloidal silica sample.

**Supplementary Figure 7-2.** Setup of the fluorescence anisotropy experiment.

Our hypothesis is that there is significant excited-state hopping in **1-PDI** that randomizes the polarization of the emitted light. If this is the case, in the presence of a quencher, which equally depletes excited states of all dipole orientations, a higher fraction of emission should occur along the original axis of excitation. In essence, the excited states that undergo more hopping events have a higher probability of having been quenched prior to emission.

**Supplementary Figure 7-3.** Hopping of the excited state randomizes emission orientation.

In accord with our prediction, the emission of the pristine **1-PDI** film appears nearly isotropic through the entire emission band. In the presence of quencher, significantly anisotropic emission is observed at the maximum.


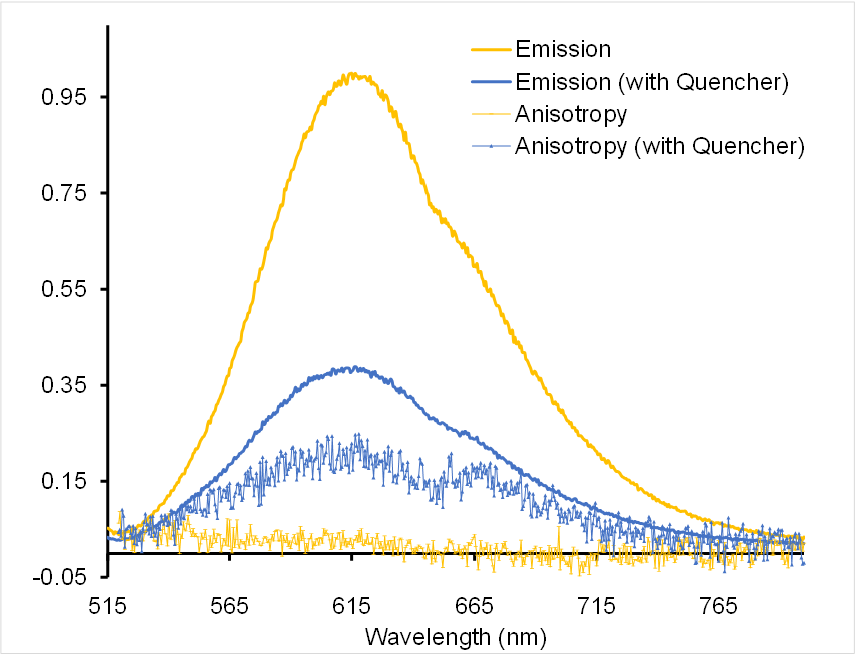


**Supplementary Figure 7-4.** Fluorescence anisotropy measurements.

## 10.3. Reversible Redox Activity

A polished quartz cuvette was filled with a dilute solution of **1-PDI** in DCM (2.0 x 10^–5^ M) and excess triethylamine (roughly 10 equiv). A cap containing a rubber septum was screwed on, and the solution was sparged with a stream of argon for 15 min. The top, including the septum, was tightly sealed with paraffin film. The solution was illuminated from the side with a single 450 nm blue LED light. UV-Vis absorbance spectra were obtained at the indicated intervals. After completion, the cap was removed, and the solution was exposed to atmospheric oxygen for 2 h.

## 10.4. Photoconductivity Measurements

A stock solution of **1-PDI** or **PDI-Mono** (1.0 mg/mL) was prepared in DCM. Using a micropipette, 6.0 μL of the solution was drop-casted on an interdigitated electrode with 200 µm gaps (CC1.WS, BVT Technologies) and dried at rt in air. The modified electrodes were submerged in a solution of 5% Et_3_N in methanol or pure methanol and the resistance of the device was measured over time (1 scan/s). Generally, the experiment involved 30 s of equilibration time (for the baseline resistance to stabilize), followed by irradiation from blue LED lamp (450 nm) for the indicated time, and subsequent recovery of baseline resistance by brief air-drying and resoaking the device in the solution. The photoreduction and aerobic oxidation cycle was repeated for at least a total of three times to ensure reproducibility and to assess reversibility. The change in resistance during this process was converted to normalized change in conductance, ΔG/G_0_, where ΔG and G_0_ are the change in conductance and baseline conductance, respectively.


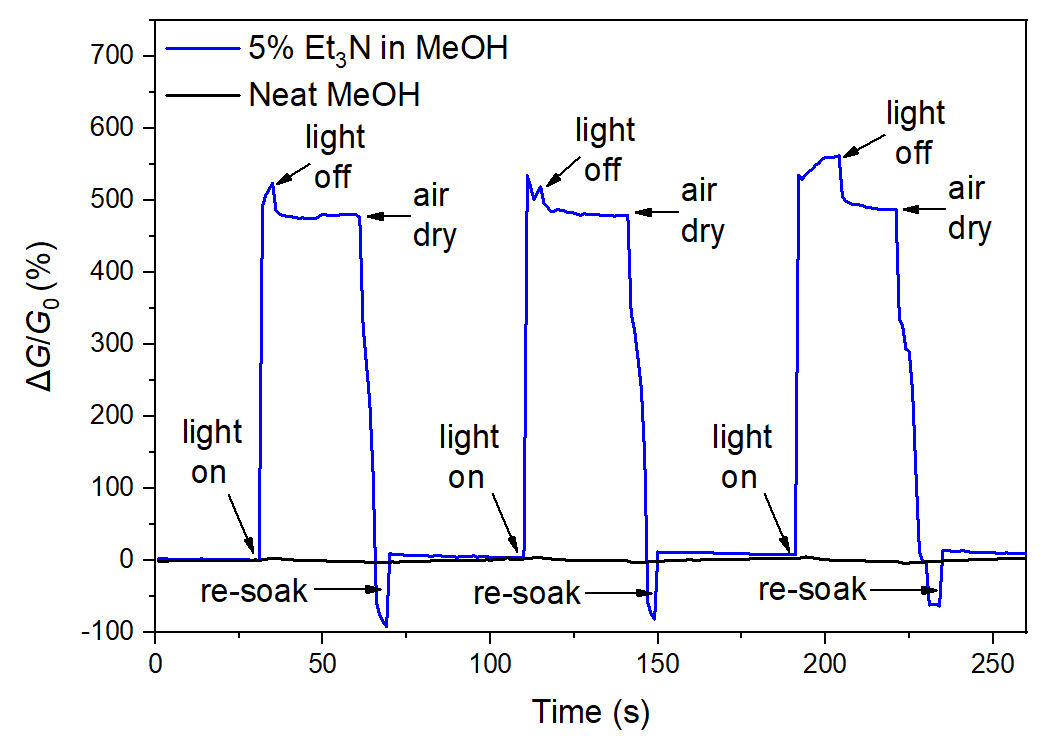

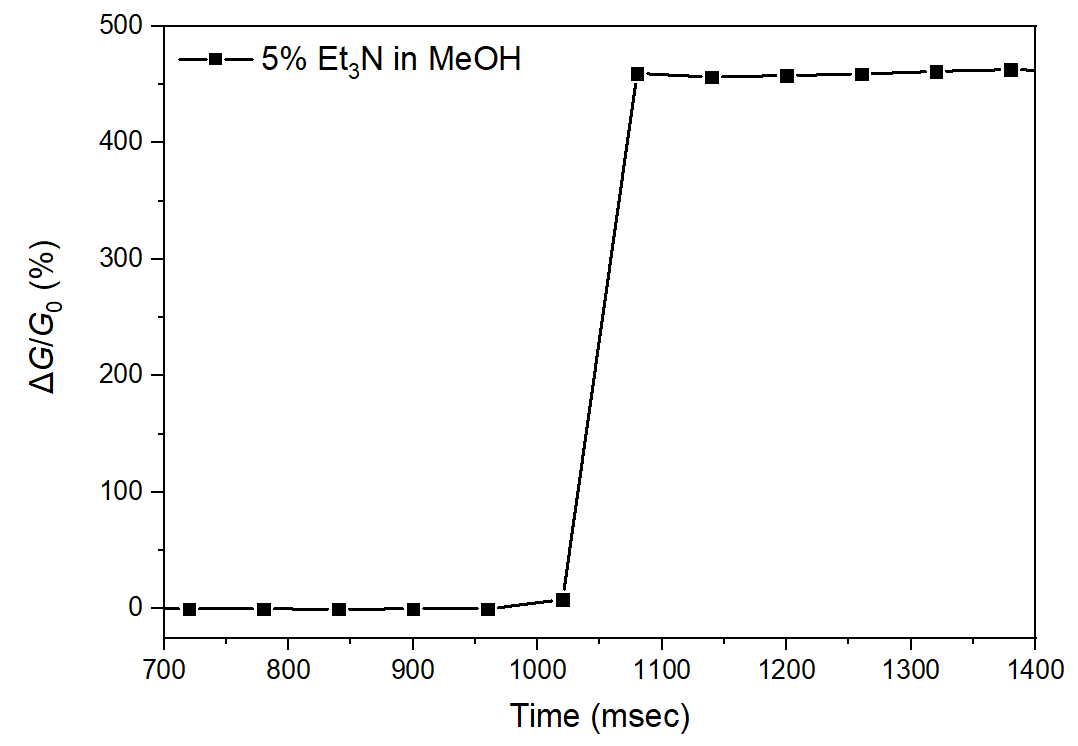


**Supplementary Figure 7-5.** (top) Normalized change in conductance for a **1-PDI** film during several cycles of photoreduction and aerobic oxidation. (bottom) Normalized change in conductance for a **1-PDI** film at the onset of irradiation. Photoactivation occurs completely within a 60 ms sampling interval.

Irradiation of a film of **1-PDI** by 450 nm light triggered a rapid increase in conductivity of *ca.* 500%, which can be attributed to the formation and facile transport of PDI radical anion units upon photo-reduction by the amine. This conductivity is maintained after the light is switched off but diminishes to the baseline value upon exposure to atmospheric oxygen, which re-oxidizes the polymer to its neutral state. As evidence that these observations reflect the mobility of charges among the PDI groups, no noticeable photo-switching was detected in the absence of an amine reductant. Further, an experiment using **PDI-Mono** as the photoconductive medium revealed similar charge transport behavior, although the rate of onset and fading of the higher-conductance state was inconsistent between cycles and often slower than for the polymer.


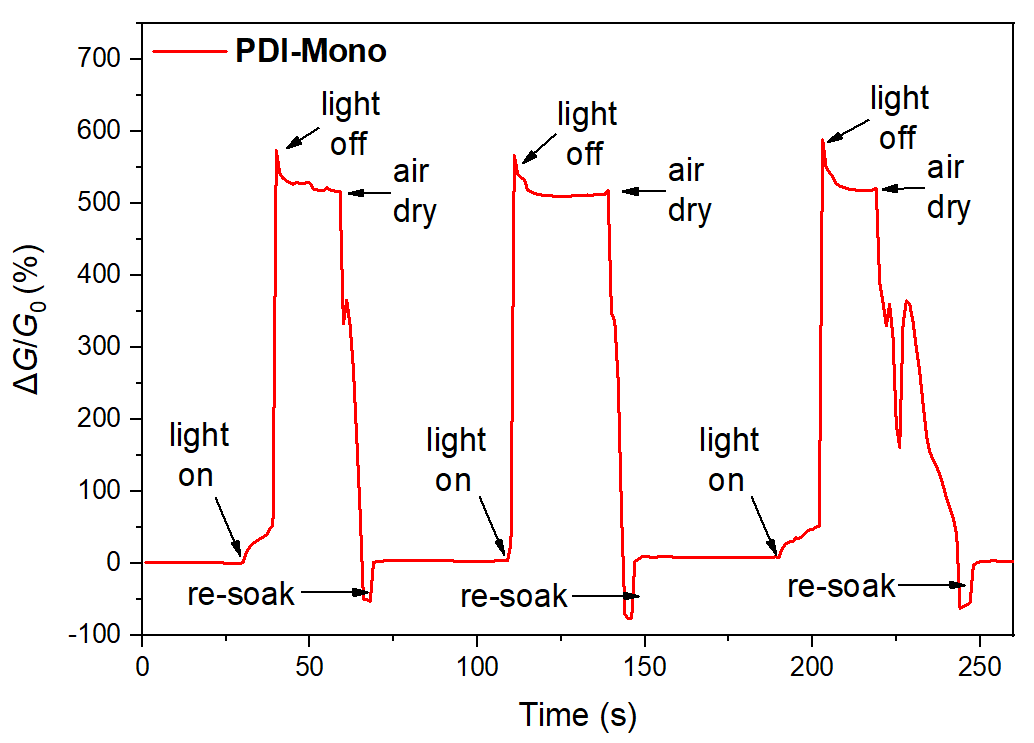


**Supplementary Figure 7-7.** Normalized change in conductance for a **PDI-Mono** film during several cycles of photoreduction and aerobic oxidation.

# Synthetic Procedures for Photoredox Catalysis

## 11.1. Experimental Procedure for Kinetic Studies

To each of four oven-dried reaction tubes, equipped with a magnetic stir bar, was added the indicated catalyst (0.090 μmol, 0.018 mol% PDI), methyl phenyl sulfide (62.1 mg, 0.50 mmol, 1.0 equiv), and methanol (2.0 mL). **1-PDI**, **1-PDI-HQ**, and **PDI-Mono** were added in powder form. For the mixture of **PDI-Mono** and **1**, the two components were first dissolved in DCM (1.0 mL) and placed into an ultrasonic bath for 10 min. The solvent was then removed with the aid of a rotary evaporator, followed by the addition of the other components as indicated above. At this point, all the vials were submerged into an ultrasonic bath for 1 min to disperse the catalysts. The vials were capped with a rubber septum, which was punctured with two 18-gauge needles to vent the reaction mixture to the atmosphere. The four vials were immersed into a LED reactor (450 nm, see below for construction) for even irradiation, and the reaction mixtures were stirred vigorously. At the indicated time-points, an aliquot (50 μL) was removed from each reaction mixture and quenched by filtration through Celite, washing with methanol (5 mL). The solvent was removed with the aid of a rotary evaporator, and the conversion was assessed by ^1^H NMR. For a 30 min period during the experiment, the reactor was turned off, leaving the mixtures to stir in the dark.

## 11.2. Synthetic Examples

**Methyl phenyl sulfoxide (3a)**

A dry 20 mL scintillation vial, equipped with a magnetic stir bar, was charged sequentially with **1-PDI** (1.34 mg, 0.18 μmol, 0.018 mol% PDI), methanol (4.0 mL), and methyl phenyl sulfide (124.2 mg, 1.0 mmol, 1.0 equiv). The vial was submerged into an ultrasonic bath for 1 min to disperse the catalyst. The vial was capped with a screw cap containing a rubber septum, which was punctured with two 18-gauge needles to vent the reaction mixture to the atmosphere. The cloudy red suspension was stirred vigorously at rt under illumination from a 450 nm blue LED lamp for 6 h. At this point, both the irradiation and the stirring were stopped, and the catalyst allowed to settle for 30 min. Additional methanol (1.0 mL) was added, and the mixture was filtered through a short plug of Celite, washing with methanol (5.0 mL). The filtrate was concentrated with the aid of a rotary evaporator, and the title compound was obtained as a clear oil (133.1 mg, 95% yield) after column chromatography on silica gel, using DCM as the eluent. The catalytic polymer **1-PDI** could be recovered from the Celite by washing with THF (10 mL). **^1^H NMR** (500 MHz, CDCl_3_) δ 7.67 – 7.61 (m, 2H), 7.55 – 7.47 (m, 3H), 2.72 (s, 3H). **^13^C NMR** (125 MHz, CDCl_3_) δ 145.9, 131.1, 129.5, 123.6, 44.1. The spectral data agreed closely with those reported in the literature.^6^

**Acetophenone (3b)**

A dry 50 mL round-bottom flask, equipped with a magnetic stir bar, was charged sequentially with 4’-bromoacetophenone (199.0 mg, 1.0 mmol, 1.0 equiv), **1-PDI** (23 mg, 0.0031 mmol, 0.31 mol% PDI), DMSO (10 mL), and triethylamine (1.39 mL, 10 mmol, 10 equiv). The flask was capped with a rubber septum and sealed tightly with paraffin film. The mixture was degassed thoroughly using four freeze-pump-thaw cycles using a needle connected to a standard Schlenk line. The cloudy red suspension was stirred vigorously at rt under illumination from 450 nm blue LED lamps for 8 h. Within the first 30 min, the mixture usually turned dark violet. After completion of the reaction, the septum was removed, and the reaction mixture was poured into a separatory funnel containing ether (50 mL) and water (50 mL). The ether phase was separated, and the aqueous phase was extracted twice more with ether (50 mL each). The organic phases were combined and washed with brine, then concentrated with the aid of a rotary evaporator. The residue was subjected to column chromatography on silica gel, using acetone as the eluent, to yield the title compound as a slightly yellow oil (118.1 mg, 99% yield). **^1^H NMR** δ 7.94 (dd, *J* = 7.8, 1.6 Hz, 2H), 7.54 (d, *J* = 7.7 Hz, 1H), 7.44 (t, *J* = 7.6 Hz, 2H), 2.58 (s, 3H). **^13^C NMR** (125 MHz, CDCl_3_) δ 198.2, 137.2, 133.2, 128.6, 128.4, 26.7. The spectral data agreed closely with those reported in the literature.^7^

**13-Bromo-1,1,1,2,2,3,3,4,4,5,5,6,6,7,7,8,8-heptadecafluoro-10-iodotridecane (3c)**

A dry, two-necked, 250 mL round-bottom flask, equipped with a large magnetic stir bar and connected to a standard Schlenk line through one neck, was charged sequentially with perfluorooctyl iodide (17.7 g, 32.5 mmol, 1.25 equiv), sodium *L*-ascorbate (1.73 g, 8.75 mmol, 0.35 equiv), **1-PDI** (92.8 mg, 0.0125 mmol, 0.05 mol% PDI), methanol (37 mL), and acetonitrile (75 mL). The open neck was capped tightly with a rubber septum, and the mixture was degassed thoroughly using three freeze-pump-thaw cycles. The flask was refilled with nitrogen, and 5-bromo-1-pentene (3.72 g, 25.0 mmol, 1.0 equiv) was added by syringe. The cloudy red suspension was stirred vigorously at rt under illumination from a 450 nm blue LED lamp until TLC analysis had confirmed disappearance of the starting iodide. After completion, the septum was carefully removed, and the mixture was diluted with water (100 mL). The crude mixture was extracted three times with DCM (100 mL each), and the combined organic phases were concentrated with the aid of a rotary evaporator. The residue was subjected to column chromatography on silica gel, using hexanes as the eluent, to yield the title compound as a clear oil (12.3 g, 65% yield), which solidified into a white foam upon drying overnight under dynamic vacuum. **Melting Point** (uncorrected): 45–47 °C. **^1^H NMR** (500 MHz, CDCl_3_) δ 4.33 (dq, *J* = 8.6, 2.7, 1.9 Hz, 1H), 3.45 (dt, *J* = 7.4, 4.3 Hz, 2H), 3.04 – 2.71 (m, 2H), 2.16 (dtd, *J* = 13.1, 7.3, 3.6 Hz, 1H), 2.06 – 1.90 (m, 3H). **^13^C NMR** (125 MHz, CDCl_3_) δ 120.1 (m), 118.1 (m), 116.0 (m), 113.3 (m), 110.8 (m), 108.5 (m), 106.2 (m), 41.9 (t, *J* = 20.9 Hz), 38.8 (d, *J* = 2.3 Hz), 32.8, 31.9, 18.9. **^19^F NMR** (470 MHz, CDCl_3_) δ -81.0 (t, *J* = 10.0 Hz), -112.0 (m), -114.5 (m), -121.6 (d, *J* = 13.2 Hz), -122.0 (dt, *J* = 27.8, 13.5 Hz), -122.8 (m), -123.7 (ddt, *J* = 23.5, 15.2, 4.2 Hz), -126.3 (m). **HRMS** (DART) calculated for C_13_H_9_BrF_17_I^+^ [M]^+^ 693.8655 amu; found 693.8660 amu. **FT-IR** (neat): 2935, 1235, 1135 cm^−1^.

**1,1-Diphenyl-2-methoxyethane (3d)**

A dry microwave vial, equipped with a magnetic stir bar, was charged sequentially with 1,1-diphenylethane (36.0 mg, 0.20 mmol, 1.0 equiv), thiophenol (11.1 mg, 0.10 mmol, 0.5 equiv), **1-PDI** (14.8 mg, 0.0020 mmol, 1.0 mol% PDI), methanol (0.75 mL), and DCM (0.25 mL). The vial was capped tightly with an aluminum lid containing a rubber septum. The mixture was degassed thoroughly using four freeze-pump-thaw cycles using a needle connected to a standard Schlenk line. The cloudy red suspension was stirred vigorously at rt under illumination from 450 nm blue LED lamps for 36 h. The reaction mixture was poured into a separatory funnel containing DCM (5 mL) and water (5 mL). The DCM phase was separated, and the aqueous phase was extracted twice more with DCM (5 mL each). The organic phases were combined and washed with brine, then concentrated with the aid of a rotary evaporator. The residue was subjected to column chromatography on silica gel, using hexanes/ethyl acetate (20:1) as the eluent, to yield the title compound as a clear oil (31.9 mg, 75% yield). **^1^H NMR** (500 MHz, CDCl_3_) δ 7.37 – 7.22 (m, 10H), 4.34 (t, *J* = 7.3 Hz, 1H), 3.97 (d, *J* = 7.3 Hz, 2H), 3.41 (s, 3H). **^13^C NMR** (125 MHz, CDCl_3_) δ 142.2, 128.6, 128.3, 126.6, 76.1, 59.0, 51.1. The spectral data agreed closely with those reported in the literature.^8^

**1,1-Diphenyl-1-methoxyethane (3d’)**

A dry microwave vial, equipped with a magnetic stir bar, was charged sequentially with 1,1-diphenylethane (36.0 mg, 0.20 mmol, 1.0 equiv), **1-Pyr** (1.2 mg, 0.0020 mmol, 1.0 mol% PDI), methanol (0.75 mL), DCM (0.25 mL), and triethylamine (50 μL). The vial was capped tightly with an aluminum lid containing a rubber septum. The mixture was degassed thoroughly using four freeze-pump-thaw cycles using a needle connected to a standard Schlenk line. The cloudy red suspension was stirred vigorously at rt under illumination from a 365 nm LED lamp for 24 h. The reaction mixture was poured into a separatory funnel containing DCM (5 mL) and water (5 mL). The DCM phase was separated, and the aqueous phase was extracted twice more with DCM (5 mL each). The organic phases were combined and washed with brine, then concentrated with the aid of a rotary evaporator. The residue was subjected to column chromatography on silica gel, using hexanes/ethyl acetate (20:1) as the eluent, to yield the title compound as a clear oil (23.4 mg, 55% yield). **^1^H NMR** (500 MHz, CDCl_3_) δ 7.49 – 7.17 (m, 10H), 3.18 (s, 3H), 1.89 (s, 3H). **^13^C NMR** (125 MHz, CDCl_3_) δ 146.5, 128.1, 126.9, 80.9, 50.7, 25.3. The spectral data agreed closely with those reported in the literature.^9^

**2-Bromo-1,3,5-trimethoxybenzene (3e)**

A dry 20 mL scintillation vial, equipped with a magnetic stir bar, was charged sequentially with 1,3,5-trimethoxybenzene (33.6 mg, 0.20 mmol, 1.0 equiv), potassium bromide (119 mg, 1.0 mmol, 5.0 equiv), **1-PDI** (4.8 mg, 6.2 μmol, 0.31 mol% PDI), DMSO (0.8 mL), and water (0.2 mL). The vial was submerged into an ultrasonic bath for 1 min to disperse the catalyst. The vial was capped with a screw cap containing a rubber septum, which was punctured with two 18-gauge needles to vent the reaction mixture to the atmosphere. The cloudy red suspension was stirred vigorously at rt under illumination from a 450 nm blue LED lamp for 36 h. At this point, both the irradiation and the stirring were stopped, and the catalyst allowed to settle for 30 min. Additional methanol (1.0 mL) was added, and the mixture was filtered through a short plug of Celite, washing with methanol (5.0 mL). The filtrate was concentrated with the aid of a rotary evaporator, and the title compound was obtained as a white powder (44.4 mg, 90% yield) after column chromatography on silica gel, using hexanes/ethyl acetate (10:1) as the eluent. **^1^H NMR** (500 MHz, CDCl_3_) δ 6.18 (s, 2H), 3.88 (s, 6H), 3.82 (s, 3H). **^13^C NMR** (125 MHz, CDCl_3_) δ 160.6, 157.6, 92.2, 91.8, 56.5, 55.7. The spectral data agreed closely with those reported in the literature.^10^

**1,3,7-Trimethyl-8-(trifluoromethyl)-3,7-dihydro-1*H*-purine-2,6-dione (3f)**

 A dry 20 mL scintillation vial, equipped with a magnetic stir bar, was charged sequentially with caffeine (38.8 mg, 0.20 mmol, 1.0 equiv), sodium trifluoromethanesulfinate (62.4 mg, 0.40 mmol, 2.0 equiv), **1-PDI** (14.8 mg, 0.0020 mmol, 1.0 mol% PDI), and DMSO (1.0 mL). The vial was submerged into an ultrasonic bath for 1 min to disperse the catalyst. The vial was capped with a screw cap, and the suspension was stirred vigorously at rt under illumination from a 450 nm blue LED lamp for 36 h. At this point, both the irradiation and the stirring were stopped, and the catalyst allowed to settle for 30 min. Additional methanol (1.0 mL) was added, and the mixture was filtered through a short plug of Celite, washing with methanol (5.0 mL). The filtrate was concentrated with the aid of a rotary evaporator, and the title compound was obtained as a white powder (37.2 mg, 71% yield) after column chromatography on silica gel, using ethyl acetate as the eluent. **^1^H NMR** (500 MHz, CDCl_3_) δ 4.16 (s, 3H), 3.59 (s, 3H), 3.42 (s, 3H). **^13^C NMR** (125 MHz, CDCl_3_) δ 155.6, 151.5, 146. 7, 139.1 (q, *J* = 40.0 Hz), 118.3 (q, *J* = 271 Hz), 109.8, 53.6, 33.3 (q, *J* = 2.3 Hz), 30.0, 28.3. **^19^F NMR** (470 MHz, CDCl_3_) δ -62.4 (s). The spectral data agreed closely with those reported in the literature.^11^

**2-(Pentafluorophenyl)-1H-pyrrole (3g)**

A dry microwave vial, equipped with a magnetic stir bar, was charged sequentially with pentafluorophenyl bromide (49.4 mg, 0.20 mmol, 1.0 equiv), potassium bicarbonate (40.0 mg, 0.40 mmol, 2.0 equiv), pyrrole (0.28 mL, 4.0 mmol, 20 equiv), **1-PDI** (14.8 mg, 0.0020 mmol, 1.0 mol% PDI), triethylamine (14.0 μL, 0.10 mmol, 0.50 equiv), and DMSO (1.0 mL). The vial was capped tightly with an aluminum lid containing a rubber septum. The mixture was degassed thoroughly using three freeze-pump-thaw cycles using a needle connected to a standard Schlenk line. The suspension was stirred vigorously at rt under illumination from a 365 nm LED lamp for 36 h. The reaction mixture was poured into a separatory funnel containing DCM (5 mL) and water (5 mL). The DCM phase was separated, and the aqueous phase was extracted twice more with DCM (5 mL each). The organic phases were combined and washed with brine, then concentrated with the aid of a rotary evaporator. The residue was subjected to column chromatography on silica gel, using hexanes/DCM (1:1) as the eluent, to yield the title compound as a white powder (25.2 mg, 54% yield). **^1^H NMR** (500 MHz, CDCl_3_) δ 9.02 (br s, 1H), 7.01 (q, *J* = 2.4 Hz, 1H), 6.91 – 6.85 (m, 1H), 6.39 (d, *J* = 3.2 Hz, 1H). **^13^C NMR** (125 MHz, CDCl_3_) δ 144.3 (m), 142.3 (m), 139.3 (m), 137.5 (m), 120.7, 117.8, 113.5 (t, *J* = 7.6 Hz), 110.3. **^19^F NMR** (470 MHz, CDCl_3_) δ -144.1 (d, *J* = 15.0 Hz), -159.4 (m), -162.5 (d, *J* = 6.6 Hz). The spectral data agreed closely with those reported in the literature.^14^

**Poly(methyl methacrylate) (4a)**

A dry microwave vial, equipped with a magnetic stir bar, was charged sequentially with ethyl 2-bromoisobutyrate (16 μL, 0.109 mmol, 1.0 equiv), methyl methacrylate (1.00 mL, 10.8 mmol, 99 equiv, distilled and passed through a plug of alumina immediately prior to use), **1-Per** (16.2 mg, 0.0023 mmol, 0.021 equiv), and DMF (1.0 mL). The vial was capped tightly with an aluminum lid containing a rubber septum. In the dark, the mixture was degassed thoroughly using five freeze-pump-thaw cycles using a needle connected to a standard Schlenk line. The suspension was placed into a water bath and stirred vigorously while exposed to natural sunlight for 6 h. Acetone (5 mL) was added, and the reaction mixture was placed into an ultrasonic bath for 1 h. The crude mixture was filtered through Celite, and the filtrate was concentrated to roughly 1 mL with the aid of a rotary evaporator. Methanol (5 mL) was added quickly, upon which the title polymer precipitated from solution as a white solid. This material was isolated by filtration, washed with additional methanol (5 mL), and dried to give the purified product (702 mg, 65% yield). Molecular weight values were calculated using ChemStation GPC Data Analysis Software (Rev. B.01.01) based on the refractive index signal. *M*n = 19.2 kg/mol, *M*w = 31.3 kg/mol, PDI = 1.6.

**4-(Octylamino)benzonitrile (4b)**

A dry microwave vial, equipped with a magnetic stir bar, was charged sequentially with 4-bromobenzonitrile (91.0 mg, 0.50 mmol, 1.0 equiv), DABCO (101 mg, 0.90 mmol, 1.8 equiv), nickel(II) bromide trihydrate (7.7 mg, 0.025 mmol, 5 mol%), octylamine (123 μL, 0.75 mmol, 1.5 equiv), **1-Bpy-Ir** (2.6 mg, 0.5 μmol, 0.10 mol%), and DMSO (1.0 mL). The vial was capped tightly with an aluminum lid containing a rubber septum. The mixture was degassed thoroughly using three freeze-pump-thaw cycles using a needle connected to a standard Schlenk line. The suspension was stirred vigorously at rt under illumination from a 450 nm LED lamp for 16 h. The reaction mixture was poured into a separatory funnel containing DCM (5 mL) and water (5 mL). The DCM phase was separated, and the aqueous phase was extracted twice more with DCM (5 mL each). The organic phases were combined and washed with brine, then concentrated with the aid of a rotary evaporator. The residue was subjected to column chromatography on silica gel, using hexanes/DCM (1:1) as the eluent, to yield the title compound as a yellow solid (104.6 mg, 91% yield). **^1^H NMR** (500 MHz, CDCl_3_) δ 7.40 (d, *J* = 8.7 Hz, 2H), 6.54 (d, *J* = 8.7 Hz, 2H), 4.22 (br s, 1H), 3.13 (t, *J* = 7.2 Hz, 2H), 1.62 (p, *J* = 7.2 Hz, 2H), 1.42 – 1.24 (m, 10H), 0.88 (t, *J* = 6.9 Hz, 3H). **^13^C NMR** (125 MHz, CDCl_3_) δ 151.6, 133.8, 120.7, 112.2, 98.4, 43.4, 31.9, 29.4, 29.3, 29.3, 27.1, 22.7, 14.2. The spectral data agreed closely with those reported in the literature.^12^

**Poly(methyl acrylate) (4a’)**

To an oven-dried pressure vessel (P260001 heavy wall, with side port, 15 mL), equipped with a stir bar, was added **1-PTZ** (4.0 mg). The vessel was evacuated, then filled with N_2_ from the Schlenk line (this process was repeated at least five times). Under nitrogen atmosphere, DMSO (1 mL), CuBr_2_ (0.5 mg, 2.2 μmol, 0.04 equiv), and PMDETA (2.3 μL, 11 μmol, 0.2 equiv) in DMSO (50 μL) were added, followed by methyl acrylate (1 mL, 11.2 mmol, 187 equiv, distilled and passed through a plug of alumina immediately prior to use) and ethyl 2-bromoisobutyrate (2.3 mg, 8.8 μL, 60 μmol, 1 equiv). The vial was sparged further with nitrogen for 2 min. The reaction tube was irradiated with a 30 W LED flood light (Ustellar IP66 400 nm). After 20 h, a small aliquot of the reaction mixture was taken and analyzed by ^1^H NMR and GPC to determine the monomer conversion and molecular weight distribution, respectively. At this point, the reaction mixture was diluted by adding 10 mL of MeCN and the photocatalyst was separated by filtration by using Durapore membrane (0.1 µm pore size, hydrophilic PVDF, 47 mm membrane). The conversion of methyl methacrylate was determined by ^1^H-NMR of the crude mixture to be 95%. Molecular-weight values were calculated using ChemStation GPC Data Analysis Software (Rev. B.01.01) based on the refractive index signal. *M*n = 14.5 kg/mol, *M*w = 15.1 kg/mol, PDI = 1.04 (Supplementary Figure 2-8).

***N*-Phenylacetamide (4c)**

To a round-bottom flask containing aniline (93 mg, 1.0 mmol, 1 equiv) in MeCN (10 mL) and **1-Acr-Me** (9.0 mg, 0.25 mol%) was added thioacetic acid (150 mg, 2.0 mmol, 2 equiv). Using a magnetic stir bar, the mixture was stirred under irradiation from a 30 W LED flood light (Ustellar IP66 400 nm) at rt for 6 h. Upon completion, the photocatalyst was separated by filtration by using Durapore membrane (0.1 µm pore size, hydrophilic PVDF, 47 mm membrane). The filtrate was diluted with water (10 mL) and then extracted with EtOAc (3×20 mL). The organic phase was washed with brine, dried over Na_2_SO_4_ and concentrated *in vacuo*. The resulting mixture was subjected to flash column chromatography (50% EtOAc in hexane) to provide pure **4c** (128 mg, 95%) as a white solid. ^1^H NMR (500 MHz, CDCl_3_) δ 7.74 (bs, 1H), 7.49 (d, J = 7.4 Hz, 2H), 7.28 (t, J = 7.9 Hz, 2H), 7.08 (t, J = 7.6 Hz, 1H), 2.16 (s, 3H). The spectral data agreed closely with those reported in the literature.^13^

## 11.3. Film Catalysis and Recycling

**Deposition of a film:** To a 20 mL scintillation vial was added **1-PDI** (2.5 mg) and THF (2.0 mL). The solid was fully dissolved using a vortexer, and the solvent was slowly removed with the aid of a rotary evaporator (130 rpm, 200 torr, rt water bath). A thin and visually homogeneous red coating was formed along roughly the bottom one-third of the vial.

**Recyclable catalysis:** To the coated vial was added sequentially methanol (2.0 mL) and methyl phenyl sulfide (62.1 mg, 0.5 mmol, 1.0 equiv). A small magnetic stir bar was added to slowly agitate the solution. Note that extremely fast stirring or too large of a stir bar can cause damage to the film, so only very slow agitation should be used (<120 rpm). An orbital mixer can also be employed. After 24 h under illumination from a 450 nm blue LED lamp, the solution was carefully transferred into a new scintillation vial. The original coated vial could be left to dry and then reused. The crude mixture was concentrated with the aid of a rotary evaporator. The yield was assessed by ^1^H NMR of the residue using CH_2_I_2_ as an internal standard. Five total cycles of this procedure were performed, producing the following yields, respectively: 99%, 96%, 99%, 100%, 99%.

**Leakage test:** One cycle of catalysis was performed as described above, and the resulting crude mixture was concentrated with the aid of a rotary evaporator. Spectroscopic grade THF (20 mL) was added to redissolve the residue, and the vial was capped shaken for 1 min to ensure full dissolution. An aliquot (1.0 mL) of this solution was diluted 100-fold and transferred to a quartz cuvette. Using a UV-Vis spectrometer, calibrated against a pure THF reference, the absorbance at 528 nm was measured, from which an estimate of the PDI content could be obtained. The absorbance value at 528 nm was less than 0.01, indicating that less than 1% PDI content was present in the output.

## 11.4. Textile Catalysis

**Preparation of the coated fabric:** a 1 in square of unbleached cotton was soaked in acetone for 30 min for cleaning. The sample was rinsed thoroughly with distilled water and dried in an oven overnight. In a 20 mL scintillation vial, **1-PDI** (10.0 mg) was combined with DCM (2.0 mL) and sonicated for 30 s to ensure full dissolution. The dye solution was applied in small batches to the cotton, drying gently with warm air in between applications. After application of dye solution (0.5 mL total per side), the fabric was dried briefly under vacuum and washed with MeOH and distilled water.

**Catalysis:** The coated cotton was placed into a 20 mL scintillation vial, along with a magnetic stir bar. To the vial was added sequentially methanol (5.0 mL) and methyl phenyl sulfide (62.1 mg, 0.5 mmol, 1.0 equiv). The reaction mixture was stirred gently under illumination from a 450 nm blue LED lamp for 24 h. The fabric was gently removed and rinsed with methanol (5.0 mL), adding the rinses to the crude reaction mixture. The crude mixture was concentrated with the aid of a rotary evaporator. The yield was assessed by ^1^H NMR of the residue using CH_2_I_2_ as an internal standard. Five total cycles of this procedure were performed, producing the following yields, respectively: 95%, 90%, 94%, 98%, 89%.

**Leakage test:** Following the procedure outlined in Section 11.3, one cycle of catalysis was performed, and the combined crude material was redissolved in THF for UV-Vis analysis. The absorbance value at 528 nm was less than 0.01, indicating that less than 1% PDI content was present in the output.

## 11.5. Magnetic Particle Catalysis

**Preparation of the coated particles:** magnetic silica particles (2 μm diameter) were purchased from Alpha Nanotech, Inc. as a 10 mg/mL aqueous dispersion. 1 mL of this dispersion was transferred to a 20 mL scintillation vial. THF (2.0 mL) was added, and the suspension was briefly mixed with a vortexer. A handheld permanent magnet was applied to the bottom of the vial to collect the magnetic particles, and roughly 90% of the solvent was decanted off. The addition of THF and decanting were repeated a total of three times to completely swap the solvent. At this point, THF (2 mL) was added, followed by **1-PDI** (5.0 mg). The suspension was mixed well and left to evaporate inside a well-ventilated fume hood to yield a dark red, fine powder. The powder was washed gently with methanol until no visible color was observed in the solution phase.

**Catalysis:** A dry 20 mL scintillation vial, equipped with a magnetic stir bar, was charged sequentially with **1-PDI**-coated magnetic nanoparticles (10 mg), methanol (2.0 mL), and methyl phenyl sulfide (62.1 mg, 0.50 mmol, 1.0 equiv). The vial was submerged into an ultrasonic bath for 1 min to disperse the catalyst. The vial was capped with a screw cap containing a rubber septum, which was punctured with two 18-gauge needles to vent the reaction mixture to the atmosphere. The cloudy red suspension placed on an orbital mixer (100 rpm) at rt under illumination from a 450 nm blue LED lamp for 6 h. At this point, both the irradiation and the stirring were stopped, and a handheld permanent magnet was applied to the bottom of the vial to collect the magnetic particles (Video S1). The methanol solution was decanted into a round-bottom flask and concentrated with the aid of a rotary evaporator. After the catalyst leakage test (see below), the crude residue was purified by column chromatography on silica gel, using DCM as the eluent to yield a clear oil (69.0 mg, 99% yield).

**Leakage test:** Following the procedure outlined in Section 11.3, the combined crude material was redissolved in THF for UV-Vis analysis. The absorbance value at 528 nm was less than 0.01, indicating that less than 1% PDI content was present in the output.

**Recovery of the catalytic coating from the magnetic particles:** The magnetic particles were added to a 20 mL scintillation vial, followed by THF (2.0 mL). After shaking the solution for a few seconds, the solution became red. A handheld permanent magnet was applied to the bottom of the vial to collect the magnetic particles (Video S2), and the red THF solution was decanted into another vial. The particles could be washed with additional THF, although we observed that very little color came into solution in subsequent washes. The light brown washed nanoparticles could be stored as a suspension in distilled water for future use.

## 11.6. Continuous-Flow Chemistry

**Coating a glass reactor coil:** A standard 14/20 reflux condenser with spiral coolant coil was used as the tubing for the flow reaction. The approximate liquid capacity of the coil was roughly 250 μL. A solution of **1-PDI** (5.0 mg) in THF (100 μL) was carefully injected into the coil. Subsequently, a rubber pipette bulb was attached to one of the inlets and used to manually shift the liquid back and forth. Simultaneously, warm water was periodically introduced into the body of the condenser to accelerate evaporation. After several minutes, the liquid had fully evaporated, and a red film was visible along the interior of the coil. Air was pumped through the coils to complete the drying process.


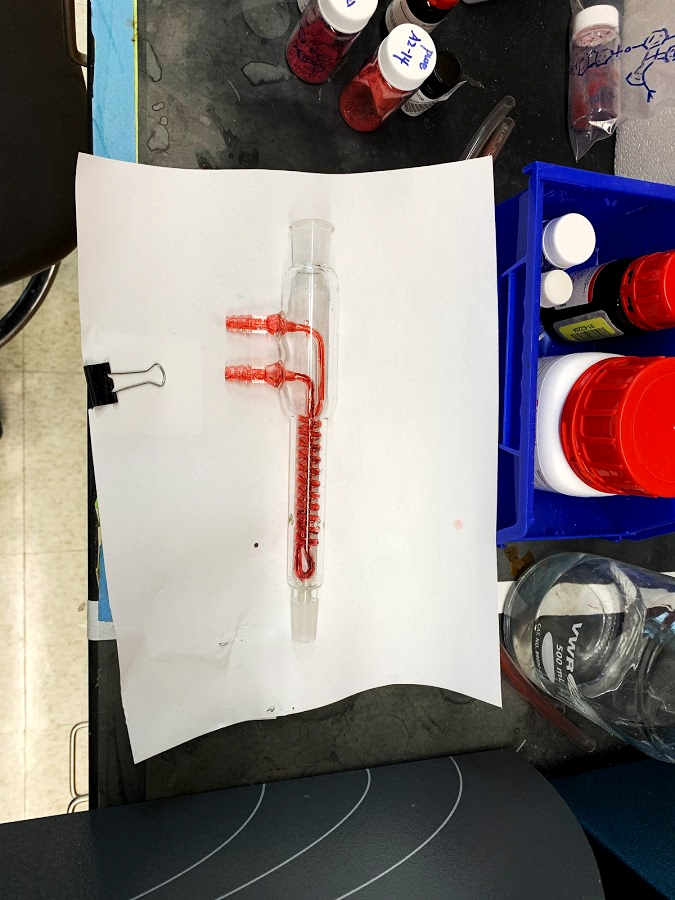


**Supplementary Figure 8-1.** Reflux condenser coated with **1-PDI**.

**Preparation of the photoreactor chamber:** LED strips (12 ft total, 18 bulbs per ft, 3.5 W per ft, 450 nm) were ordered from 1000Bulbs.com and installed along the inside of a 1 L glass beaker. The exterior of the beaker was covered with aluminum foil. The lights were powered by a 60 W power supply.


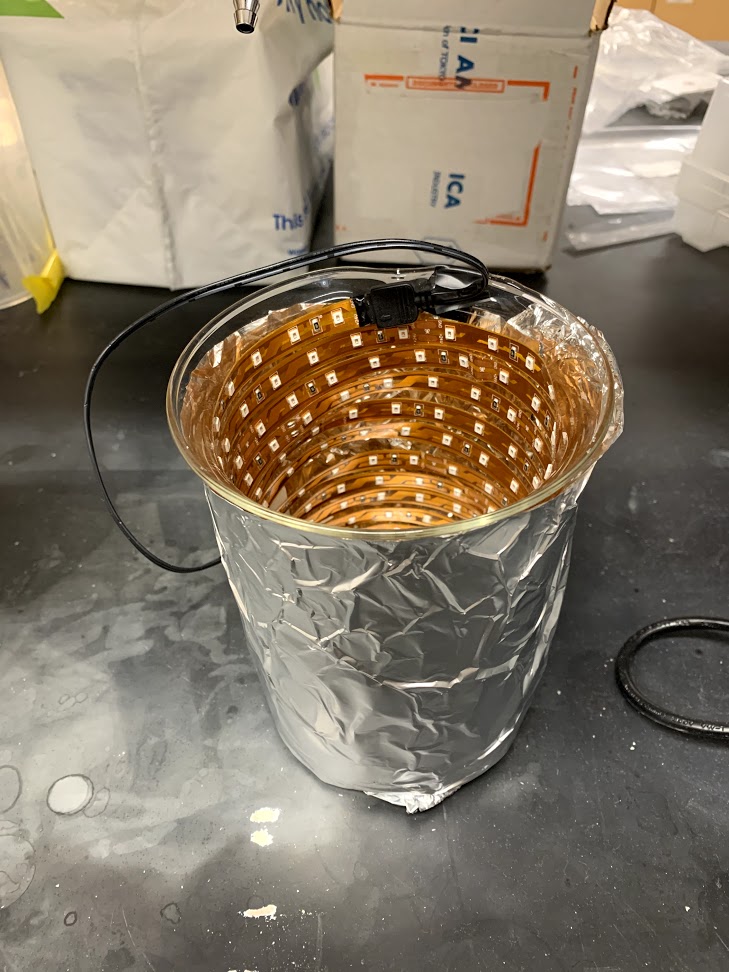

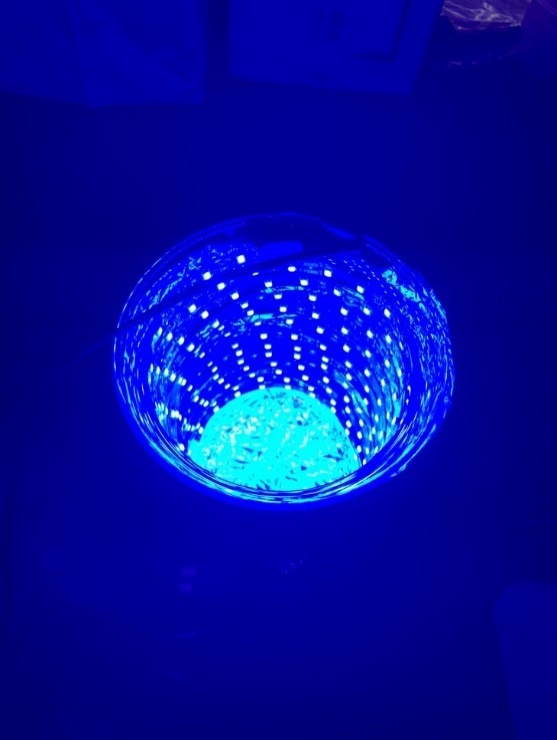


**Supplementary Figure 8-2.** Photoreactor used for flow chemistry.

**Catalytic reaction in glass flow apparatus:** A glass syringe was filled with a solution containing 1,3,5-trimethoxybenzene (326 mg, 2.0 mmol, 1.0 equiv), potassium bromide (476 mg, 4.0 mmol, 2.0 equiv), ammonium persulfate (456 mg, 2.0 mmol, 1.0 equiv), DMSO (16.0 mL), and water (4.0 mL). The solution was passed through the reactor coil at a rate of 250 μL/h. The condenser was submerged into the photoreactor, and compressed air was run through the body of the condenser for cooling. After 1 h of equilibration time, an aliquot was collected for 1 h, and the yield/conversion was assessed by ^1^H NMR analysis, indicating 68% yield.

**Leakage test:** Following the procedure outlined in Section 11.3, after NMR analysis, the aliquot was redissolved in THF for UV-Vis analysis. The absorbance value at 528 nm was less than 0.01, indicating that less than 1% PDI content was present in the output.


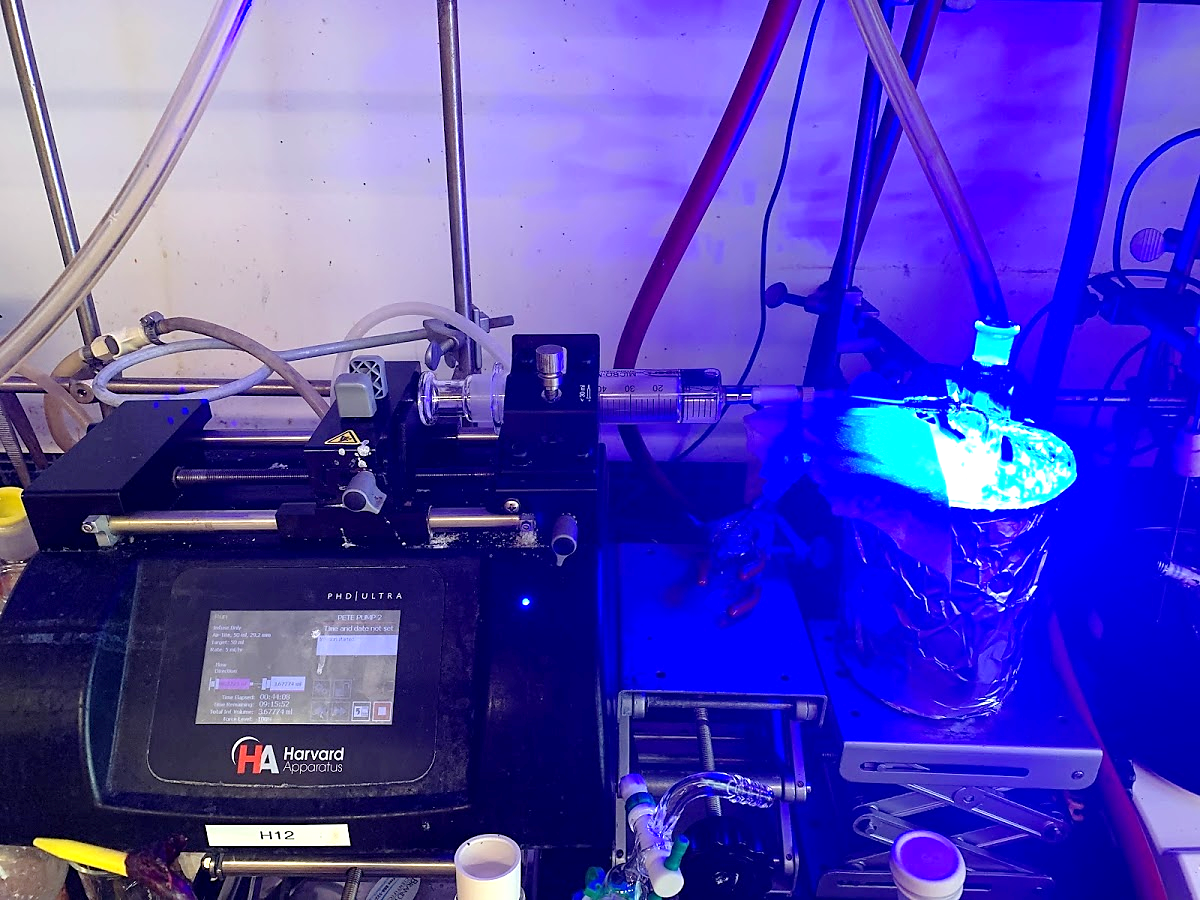


**Supplementary Figure 8-3.** Continuous-flow photoredox setup.

**Coating of fluorinated tubing with catalytic polymer:** Flexible tubing made from Dupont perfluoroalkoxyalkane (PFA) polymer was obtained from Upchurch Scientific (1509L), with inner diameter of 0.062 in (1.55 mm) and exterior diameter of 0.125 in. Using a syringe, a solution of **1-PDI** (5.0 mg) or **1-PDI-C_17_F_35_** (5.0 mg) in DCM (100 μL) was carefully injected into a 60 cm segment of tubing. By tilting, the red solution was moved back and forth slowly within the tubing. This process was carried out in a well-ventilated fume hood. Occasional submersion in a gently warmed water bath could accelerate evaporation, although heating above the boiling point of DCM caused the formation of bubbles, which should be avoided. As the solvent was almost fully evaporated, the tubing was held straight and fully vertical, reversing direction roughly every 10 seconds. When no more liquid motion was apparent, the tubing was left to air-dry overnight, followed by further drying with a very slow stream of air. While the coating process was successful with **1-PDI-C_17_F_35_**, we were unable to achieve the visually uniform layer using **1-PDI**, which tended to crystallize near the end of the process to form powder deposits that often clogged the tubing. Once the PFA tubing had been successfully coated, the quality could be tested by flowing pure MeOH or H_2_O by syringe pump at 0.100 mL/min for 30 min. The collected output should contain no red polymer flakes, and when redissolved in DCM, should not have any red color. The coated segment of the tubing was about 45 cm in length, or 850 μL in volume.


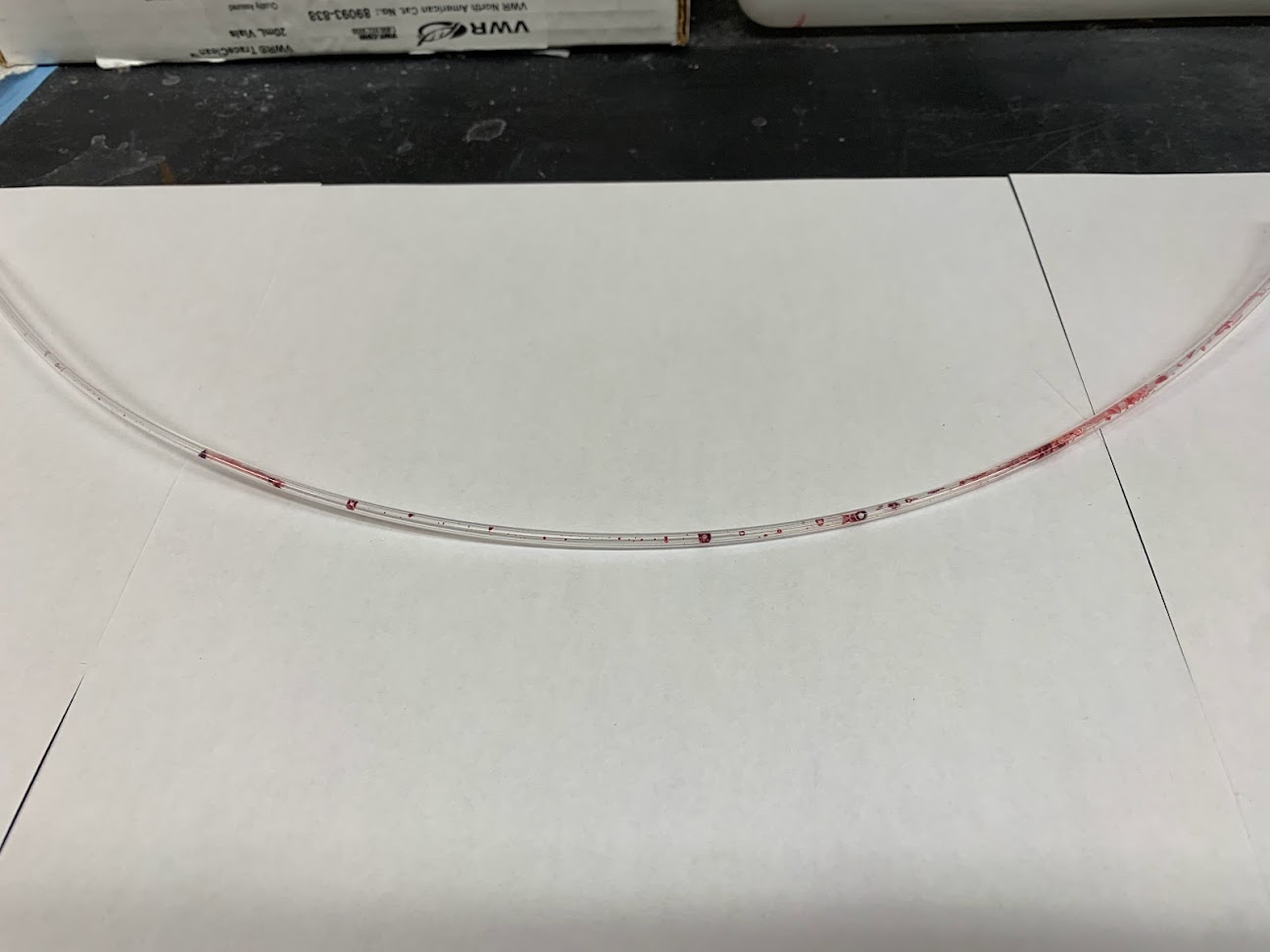


**Supplementary Figure 8-4.** Attempted coating of PFA tubing with **1-PDI**.


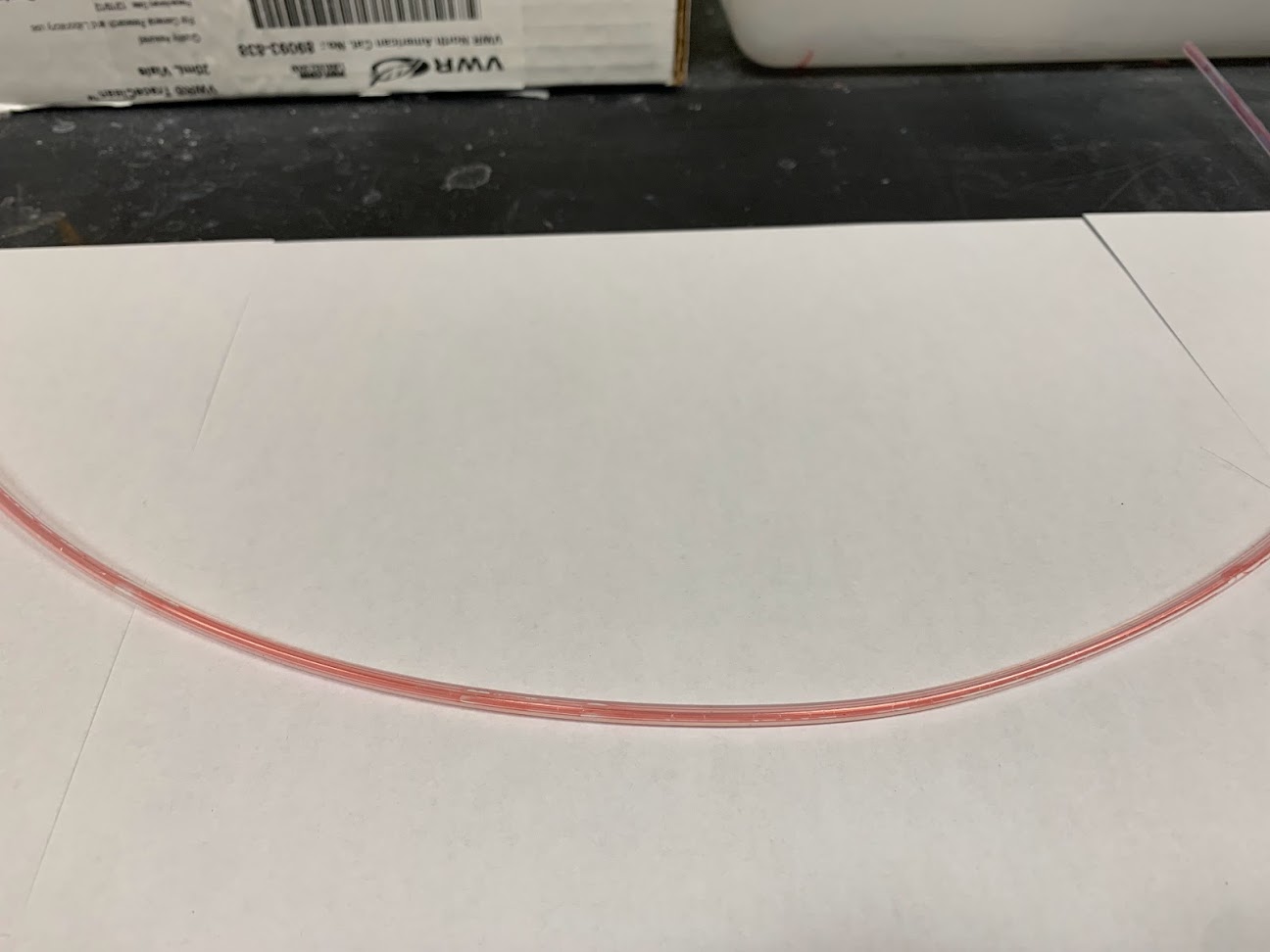


**Supplementary Figure 8-5.** Coating of PFA tubing with perfluoroalkylated **1-PDI-C_17_F_35_**.

**Catalytic reaction in fluorinated plastic flow apparatus:** The PFA tubing, coated with **1-PDI-C_17_F_35_**, was wrapped carefully around a 250 mL beaker. Using a two-sided adaptor, one end was connected to a glass syringe operated by an automated syringe pump. The other end of the reactor was joined to a piece of connector tubing leading into a 20 mL collection vial. The glass syringe was filled with a solution containing 1,3,5-trimethoxybenzene (326 mg, 2.0 mmol, 1.0 equiv), potassium bromide (476 mg, 4.0 mmol, 2.0 equiv), ammonium persulfate (456 mg, 2.0 mmol, 1.0 equiv), DMSO (16.0 mL), and water (4.0 mL). The solution was passed through the reactor coil at a rate of 28.3 μL/min. The coil of tubing was submerged into the photoreactor shown in Supplementary Figure 8-2, and a fan was directed into the photoreactor for cooling. After 1 h of equilibration time, an aliquot was collected for 30 min, and the yield/conversion was assessed by ^1^H NMR analysis, indicating 64% yield.

# NMR Spectra of Photoredox Products

**^1^H NMR** (500 MHz, CDCl_3_)

**Supplementary Figure 12-1.** ^1^H NMR spectrum of **3a**.

**^13^C NMR** (125 MHz, CDCl_3_)

**Supplementary Figure 12-2.** ^13^C NMR spectrum of **3a**.

**^1^H NMR** (500 MHz, CDCl_3_)

**Supplementary Figure 12-3.** ^1^H NMR spectrum of **3b**.

**^13^C NMR** (125 MHz, CDCl_3_)

**Supplementary Figure 12-4.** ^13^C NMR spectrum of **3b**.

**^1^H NMR** (500 MHz, CDCl_3_)

**Supplementary Figure 12-5.** ^1^H NMR spectrum of **3c**.

**^13^C NMR** (125 MHz, CDCl_3_)

**Supplementary Figure 12-6.** ^13^C NMR spectrum of **3c**.

**^19^F NMR** (470 MHz, CDCl_3_)

**Supplementary Figure 12-7.** ^19^F NMR spectrum of **3c**.

**^1^H NMR** (500 MHz, CDCl_3_)

**Supplementary Figure 12-8.** ^1^H NMR spectrum of **3d**.

**^13^C NMR** (125 MHz, CDCl_3_)

**Supplementary Figure 12-9.** ^13^C NMR spectrum of **3d**.

**^1^H NMR** (500 MHz, CDCl_3_)

**Supplementary Figure 12-10.** ^1^H NMR spectrum of **3d’**.

**^13^C NMR** (125 MHz, CDCl_3_)

**Supplementary Figure 12-11.** ^13^C NMR spectrum of **3d’**.

**^1^H NMR** (500 MHz, CDCl_3_)

**Supplementary Figure 12-12.** ^1^H NMR spectrum of **3e**.

**^13^C NMR** (125 MHz, CDCl_3_)

**Supplementary Figure 12-13.** ^13^C NMR spectrum of **3e**.

**^1^H NMR** (500 MHz, CDCl_3_)

**Supplementary Figure 12-14.** ^1^H NMR spectrum of **3f**.

**^13^C NMR** (125 MHz, CDCl_3_)

**Supplementary Figure 12-15.** ^13^C NMR spectrum of **3f**.

**^19^F NMR** (470 MHz, CDCl_3_)

**Supplementary Figure 12-16.** ^19^F NMR spectrum of **3f**.

**^1^H NMR** (500 MHz, CDCl_3_)

**Supplementary Figure 12-17.** ^1^H NMR spectrum of **3g**.

**^13^C NMR** (125 MHz, CDCl_3_)

**Supplementary Figure 12-18.** ^13^C NMR spectrum of **3g**.

**^19^F NMR** (470 MHz, CDCl_3_)

**Supplementary Figure 12-19.** ^19^F NMR spectrum of **3g**.

**^1^H NMR** (500 MHz, CDCl_3_)

**Supplementary Figure 12-20.** ^1^H NMR spectrum of **4b**.

**^13^C NMR** (125 MHz, CDCl_3_)

**Supplementary Figure 12-21.** ^13^C NMR spectrum of **4b**.

# Supplementary References

(1) Yang, J.-S., Swager, T. M. Fluorescent porous polymer films as TNT chemosensors: electronic and structural effects. *J. Am. Chem. Soc.* **120**, 11864‒11873 (1998).

(2) Zhu, Z., Swager, T. M. Conjugated polymer liquid crystal solutions: control of conformation and alignment. *J. Am. Chem. Soc.* **124**, 9670‒9671 (2002).

(3) Tahara, T. *et al.* Triplet diradical-cation salts consisting of the phenothiazine radical cation and a nitronyl nitroxide. *Chem. Eur. J.* **25**, 7201‒7209 (2019)

(4) Mizoshita, N., Yamanaka, K.-I., Shimada, T., Tani, T., Inagaki, S. Mesostructured organosilica with a 9-mesityl-10-methylacridinium bridging unit: photoinduced charge separation in the organosilica framework. *Chem. Commun.* **46**, 9235‒9237 (2010).

(5) Yin, D., Su, D., Jin, J. Photoredox catalytic trifluoromethylation and perfluoroalkylation of arenes using trifluoroacetic and related carboxylic acids. *Cell Rep. Phys. Sci.* **1**, 100141 (2020).

(6) Danahy, K. E., Styduhar, E. D., Fodness, A. M., Heckman, L. M., Jamison, T. F. On-demand generation and use in continuous synthesis of the ambiphilic nitrogen source chloramine. *Org. Lett.* **22**, 8392‒8395 (2020).

(7) Modak, A. *et al.* A general and efficient aldehyde decarbonylation reaction by using a palladium catalyst. *Chem. Commun.* **48**, 4253‒4255 (2012).

(8) Yi, H. *et al.* Photocatalytic dehydrogenative cross-coupling of alkenes with alcohols or azoles without external oxidant. *Angew. Chem. Int. Ed.* **56**, 1120‒1124 (2006).

(9) Mangion, D., Arnold, D. R. The photochemistry of 4-halobenzonitriles and 4-haloanisoles with 1,1-diphenylethene in methanol. Homolytic cleavage versus electron-transfer pathways. *Can. J. Chem.* **77**, 1655‒1670 (1999).

(10) Li, Z. *et al.* Additive-free copper(I)-mediated synthesis of 5- or 6-brominated 2-aryl-1*H*-indole-3-carboxylates from α,α-dibromo β-iminoesters. *J. Org. Chem.* **86**, 1964‒1971 (2021).

(11) O'Brien, A. G. *et al.* Radical C‒H functionalization of heteroarenes under electrochemical control. *Angew. Chem. Int. Ed.* **53**, 11868‒11871 (2014).

(12) Ogata, T., Hartwig, J. F. Palladium-catalyzed amination of aryl and heteroaryl tosylates at room temperature. *J. Am. Chem. Soc.* **130**, 13848‒13849 (2008).

(13) Mali, S. M., Bhaisare, R. D., Gopi, H. N. Thioacids mediated selective and mild *N*-acylation of amines. *J. Org. Chem.* **78**, 5550‒5555 (2013).

(14) Ghosh, I. et al. Organic semiconductor photocatalyst can bifunctionalize arenes and heteroarenes. *Science* **365**, 360–366 (2019).
