# Supplementary material for: Solution-processable microporous polymer platform for heterogenization of diverse photoredox catalysts
Source: Nat Commun. 2022 May 27;13:2775. doi: 10.1038/s41467-022-29811-6 (PMC9142596; doi:10.1038/s41467-022-29811-6)
Supplement: Supplementary file 2 — Description of Additional Supplementary Files [file 41467_2022_29811_MOESM2_ESM.pdf]

Description of Additional Supplementary files

File name: Supplementary Movie 1

Description: Magnetic separation of catalyst from acetonitrile solution.

File name: Supplementary Movie 2

Description: Desorption and separation of 1-PDI from magnetic nanoparticles using tetrahydrofuran.
